# Photoreceptor protection by mesenchymal stem cell transplantation identifies exosomal MiR-21 as a therapeutic for retinal degeneration

**DOI:** 10.1038/s41418-020-00636-4

**Published:** 2020-10-20

**Authors:** Chun-Lei Deng, Cheng-Biao Hu, Sheng-Tao Ling, Na Zhao, Li-Hui Bao, Feng Zhou, Ye-Cheng Xiong, Tao Chen, Bing-Dong Sui, Xiao-Rui Yu, Cheng-Hu Hu

**Affiliations:** 1 Department of Biochemistry and Molecular Biology, School of Basic Medical Sciences, Xi’an Jiaotong University, Xi’an, 710061 Shaanxi China; 2 Xi’an Institute of Tissue Engineering and Regenerative Medicine, Xi’an, 710032 Shaanxi China; 3 Central Laboratory, Taihe Hospital affiliated to Xi’an Jiaotong University, Shiyan, 442000 Hubei China; 4 State Key Laboratory of Military Stomatology & National Clinical Research Center for Oral Diseases & Shaanxi International Joint Research Center for Oral Diseases, Center for Tissue Engineering, School of Stomatology, The Fourth Military Medical University, Xi’ an, 710032 Shaanxi China

**Keywords:** Epigenetics, Diseases, Stem-cell research, Translational research

## Abstract

Photoreceptor apoptosis is recognized as one key pathogenesis of retinal degeneration, the counteraction of which represents a promising approach to safeguard visual function. Recently, mesenchymal stem cell transplantation (MSCT) has demonstrated immense potential to treat ocular disorders, in which extracellular vesicles (EVs), particularly exosomes, have emerged as effective ophthalmological therapeutics. However, whether and how MSCT protects photoreceptors against apoptotic injuries remains largely unknown. Here, we discovered that intravitreal MSCT counteracted photoreceptor apoptosis and alleviated retinal morphological and functional degeneration in a mouse model of photoreceptor loss induced by *N*-methyl-*N*-nitrosourea (MNU). Interestingly, effects of MSCT were inhibited after blockade of exosomal generation by GW4869 preconditioning. Furthermore, MSC-derived exosomal transplantation (EXOT) effectively suppressed MNU-provoked photoreceptor injury. Notably, therapeutic efficacy of MSCT and EXOT on MNU-induced retinal degeneration was long-lasting as photoreceptor preservance and retinal maintenance were detected even after 1–2 months post to injection for only once. More importantly, using a natural occurring retinal degeneration model caused by a nonsense mutation of *Phosphodiesterase 6b* gene (*Pde6b*^*mut*^), we confirmed that MSCT and EXOT prevented photoreceptor loss and protected long-term retinal function. In deciphering therapeutic mechanisms regarding potential exosome-mediated communications, we identified that miR-21 critically maintained photoreceptor viability against MNU injury by targeting programmed cell death 4 (Pdcd4) and was transferred from MSC-derived exosomes in vivo for functional regulation. Moreover, miR-21 deficiency aggravated MNU-driven retinal injury and was restrained by EXOT. Further experiments revealed that miR-21 mediated therapeutic effects of EXOT on MNU-induced photoreceptor apoptosis and retinal dysfunction. These findings uncovered the efficacy and mechanism of MSCT-based photoreceptor protection, indicating exosomal miR-21 as a therapeutic for retinal degeneration.

## Introduction

Photoreceptor apoptosis is recognized as one of the major form of photoreceptor death and one important contributor to visual loss in retinal degenerative disorders [1, 2], such as inherited retinal dystrophies encompassing the retinitis pigmentosa (RP) [3]. Recently, mesenchymal stem cell transplantation (MSCT) has demonstrated immense potential to ameliorate a variety of ocular dysfunctions [4, 5]. Particularly, MSCT retards retinal degeneration with the prevention of loss of retinal ganglion cells (RGCs) and retinal pigment epithelium (RPE) [6–9]. However, despite evidence indicating that MSCT rescues photoreceptor deficiency in mice with genetic defects [9–11], it remains unknown whether MSCT is efficient in directly protecting photoreceptors against specific cell death stimuli in vivo, e.g., light exposure and certain phototoxins [12, 13]. Elucidating potential effects and underlying mechanisms of MSCT counteracting photoreceptor apoptosis would therefore provide promise for establishing novel therapeutics of retinal degeneration.

Emerging evidence suggests that paracrine effects of MSCT to create beneficial microenvironments for functional recovery of recipient cells represent the crucial mechanisms underlying MSC-based disease therapeutics [14, 15]. Correspondingly, subretinal or intravitreal transplanted MSCs exert profound trophic effects on the retina, which constitute soluble neuroprotective cytokines [8, 16] and extracellular vesicles (EVs) carrying retina-regulatory molecules [17, 18]. In particular, intravitreal delivery of MSC-secreted exosomes, EVs of endocytic origin with less than 100 nm diameter in size [19], has been reported to promote survival of RGCs after traumatic optic neuropathy [17]. Notably, exosomes are increasingly documented as physiological regulators of retinal homeostasis [20] and important indicators of retinopathies [21], including being involved in photoreceptor degeneration upon insufficient release [22]. Nevertheless, whether exosomal transplantation (EXOT) shows efficacy to ameliorate photoreceptor loss and improve degenerative retinopathies has not yet been evaluated.

It is widely accepted that EVs mediate intercellular communications through exchange of proteins, mRNAs, and mostly the microRNAs, which are ~22-nucleotide-long noncoding RNAs that negatively regulate expression of target genes at the posttranscriptional level [23]. Especially, exosomal transfer of microRNAs has been extensively reported to mediate effects of MSCT in treating a range of disorders [24–26]. In this regard, studies have identified individual microRNAs that play important roles in regulating ocular and retinal cell function, including those essential for photoreceptor maturation, maintenance, and visual function [27–29]. Furthermore, microRNA dysregulation has been revealed to be associated with photoreceptor death in the degenerating retina [30, 31]. Importantly, a pioneer study has reported diminished effects of MSC-derived exosomes on preventing traumatic loss of RGCs after knockdown of Argonaute-2, a key microRNA effector molecule, suggesting microRNA-dependent mechanisms for retinal protection [17]. However, there is still a lack of data unraveling specific microRNA(s) that regulates photoreceptor apoptosis and supports retinal therapeutics.

In this study, we aimed to investigate whether MSCT protects photoreceptors against apoptosis in counteracting retinal degeneration, and whether exosomal microRNA transfer serves as the therapeutic mechanism.

## Results

### Intravitreal MSCT counteracts MNU-induced photoreceptor apoptosis and alleviates retinal degeneration

**Fig. 1:**
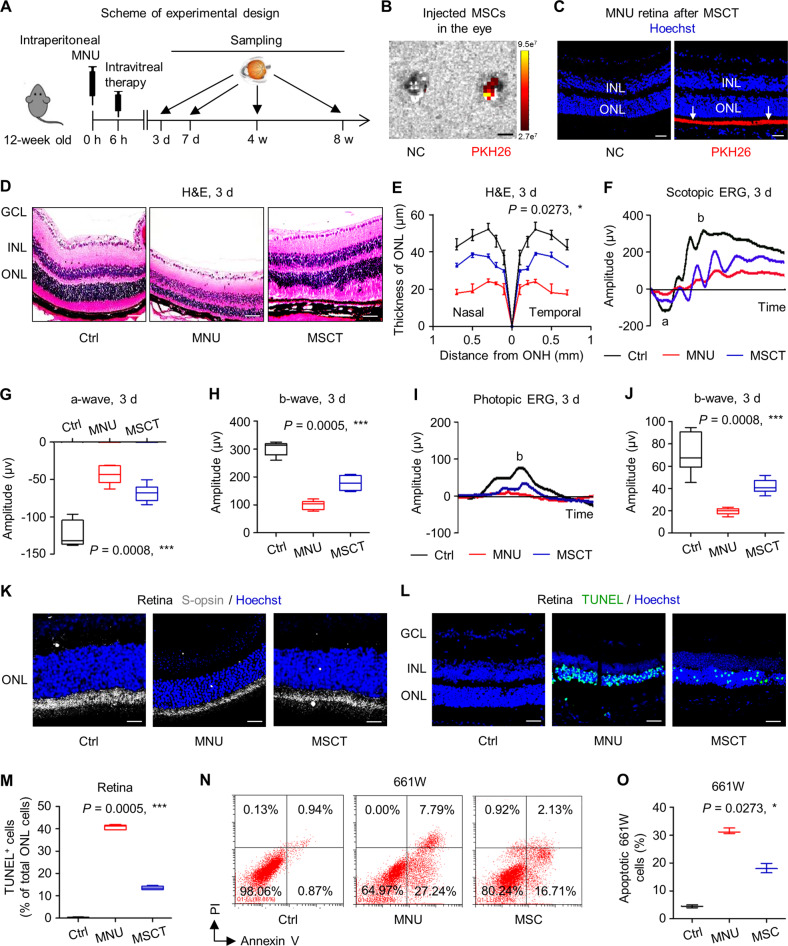
Intravitreal transplantation of mesenchymal stem cells (MSCs) counteracts *N*-methyl-*N*-nitrosourea (MNU)-induced photoreceptor apoptosis and alleviates retinal degeneration. **A** Schematic diagram demonstrating the study design of in vivo experiments on MNU-induced retinal degeneration. **B** Biodistribution of PKH26-labeled MSCs in the eye after intravitreal injection for 6 h. NC negative control, injection of MSCs without staining. Scale bar = 2.5 mm. **C** Tracing of PKH26-labeled MSCs (red) in the retina tissue counterstained by Hoechst 33342 (blue) after intravitreal injection for 24 h. MSCT mesenchymal stem cell transplantation after MNU injection, INL inner nuclear layer, ONL outer nuclear layer. Scale bar = 50 μm. Representative hematoxylin and eosin (H&E) staining images of retinal tissues (**D**) and the corresponding quantitative analysis of ONL thickness (**E**). Ctrl control, no MNU treatment, GCL ganglion cell layer, ONH optic nerve head. Scale bars = 50 μm. *P < 0.05 by the Kruskal–Wallis test for area under curve (AUC). *N* = 3 per group. Representative scotopic electroretinography (ERG) waveforms (**F**) and the corresponding quantitative analyses of amplitude changes of a-wave (**G**) and b-wave (**H**). *P < 0.05 by the Kruskal–Wallis tests. *N* = 6 per group. Representative photopic ERG waveforms (**I**) and the corresponding quantitative analysis of b-wave amplitude changes (**J**). *P < 0.05 by the Kruskal–Wallis tests. *N* = 6 per group. **K** Representative immunofluorescent (IF) staining images of retinal tissues showing cone photoreceptor bodies (white) counterstained by Hoechst 33342 (blue). Scale bars = 25 μm. Representative terminal deoxynucleotidyl transferase dUTP nick end labeling (TUNEL, green) staining images of retinal tissues counterstained by Hoechst 33342 (blue) (**L**) and the corresponding quantitative analysis of percentages of TUNEL^+^ cells over total ONL cells (**M**). Scale bars = 50 μm. *P < 0.05 by the Kruskal–Wallis test. *N* = 6 per group. Representative flow cytometric images showing death of 661W cone photoreceptor cells (**N**) and the corresponding quantitative analysis of percentages of apoptotic (Annexin V^+^PI^−^ plus Annexin V^+^PI^+^) 661W cells (**O**). The MSC group, Transwell co-culture of MNU-treated 661W cells with MSCs. PI propidium iodide. *P < 0.05 by the Kruskal–Wallis test. *N* = 3 per group. Data represent median ± range for (**E**) and (**O**). Data are represented as box (25th, 50th, and 75th percentiles) and whisker (range) plots for (**G**), (**H**), (**J**), and (M).

Allogeneic MSCs applied in this study was isolated from mouse bone marrow and was confirmed for their identity and suitability for therapeutic usage according to current criteria (Fig. S1A–C) [32, 33]. To investigate whether MSCT serves as an effective therapeutic for photoreceptor loss in retinal degeneration, the phototoxin *N*-methyl-*N*-nitrosourea (MNU) was used to establish a photoreceptor-specific injury model in mice [13], and MSCs were delivered intravitreally (Fig. 1A). Biodistribution analyses confirmed that PKH26-labeled MSCs engrafted in the eye with specific location in the photoreceptor-nuclei-residing outer nuclear layer (ONL) (Fig. 1B, C).

As expected, intraperitoneal injection of MNU-triggered remarkable degeneration of the retinal tissue after 3 d, in which the ONL underwent substantial loss (Fig. 1D, E). Importantly, intravitreal MSCT significantly counteracted detrimental effects of MNU, leading to preserved integrity of retinal tissues with improved ONL thickness at 3 d (Fig. 1D, E). To further examine function of photoreceptors, we performed electroretinography (ERG) analyses, in which scotopic (dark-adapted, mixed rod- and cone-mediated) and photopic (light-adapted, cone-mediated) responses are both recorded for depicting a-wave and/or b-wave, respectively reflecting hyperpolarization of photoreceptors and the subsequent depolarization of bipolar cells postsynaptic to photoreceptors [34, 35]. As detected, scotopic ERG analyses demonstrated protection of MSCT on the retinal electrophysiological function against MNU damages, quantified by higher amplitudes of both a-wave and b-wave (Fig. 1F–H). Also, photopic ERG responses were significantly protected by MSCT (Fig. 1I, J), which was confirmed by S-opsin staining exhibiting ameliorated loss of cone photoreceptors by MSCT against MNU (Fig. 1K). Terminal deoxynucleotidyl transferase dUTP nick end labeling (TUNEL) analysis further showed that therapeutic effects of MSCT on MNU-provoked retinal damages were based on inhibition of the apoptosis of photoreceptors in the ONL (Fig. 1L, M). As verified by in vitro experiments, MNU-induced apoptosis of the 661W cone photoreceptor cell line and that indirect co-culture of MSCs with 661W cells in the Transwell system counteracted detrimental effects of MNU, which also indicated paracrine mechanisms (Fig. 1N, O). Protective effects of MSCT on MNU-treated retina were continuously prominent at 7 d, when both morphological and electrophysiological alterations were dramatically restrained (Fig. S2A–G). These findings suggested that intravitreal MSCT effectively counteracts MNU-induced photoreceptor apoptosis and alleviates retinal degeneration.

### Exosomes mediates therapeutic effects of MSCT on MNU-provoked retinal photoreceptor damages

**Fig. 2:**
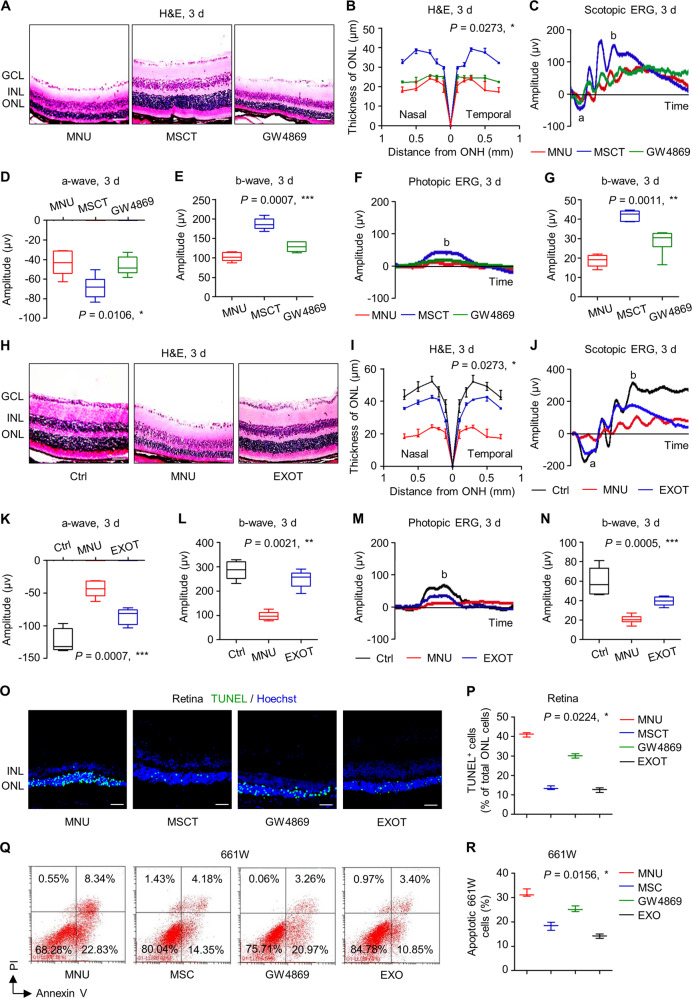
Exosomes mediates therapeutic effects of mesenchymal stem cell transplantation (MSCT) on *N*-methyl-*N*-nitrosourea (MNU)-induced photoreceptor apoptosis and retinal degeneration. Representative hematoxylin and eosin (H&E) staining images of retinal tissues (**A**) and the corresponding quantitative analysis of outer nuclear layer (ONL) thickness (**B**). Mice were injected with MNU with or without MSCT. GW4869, a neutral sphingomyelinase inhibitor for blocking exosome generation, which was used for MSC preconditioning before transplantation; GCL ganglion cell layer, INL inner nuclear layer, ONH optic nerve head. Scale bars = 50 μm. *P < 0.05 by the Kruskal–Wallis test for area under curve (AUC). *N* = 3 per group. Representative scotopic electroretinography (ERG) waveforms (**C**) and the corresponding quantitative analyses of amplitude changes of a-wave (**D**) and b-wave (**E**). *P < 0.05 by the Kruskal–Wallis tests. *N* = 6 per group. Representative photopic ERG waveforms (**F**) and the corresponding quantitative analysis of b-wave amplitude changes (**G**). *P < 0.05 by the Kruskal–Wallis tests. *N* = 6 per group. Representative H&E staining images of retinal tissues (**H**) and the corresponding quantitative analysis of ONL thickness (**I**). Ctrl control, no MNU treatment, EXOT exosomal transplantation after MNU injection. Scale bars = 50 μm. *P < 0.05 by the Kruskal–Wallis test for AUC. *N* = 3 per group. Representative scotopic ERG waveforms (**J**) and the corresponding quantitative analyses of amplitude changes of a-wave (**K**) and b-wave (**L**). *P < 0.05 by the Kruskal–Wallis tests. *N* = 6 per group. Representative photopic ERG waveforms (**M**) and the corresponding quantitative analysis of b-wave amplitude changes (**N**). *P < 0.05 by the Kruskal–Wallis tests. *N* = 6 per group. Representative terminal deoxynucleotidyl transferase dUTP nick end labeling (TUNEL, green) staining images of retinal tissues counterstained by Hoechst 33342 (blue) (**O**) and the corresponding quantitative analysis of percentages of TUNEL^+^ cells over total ONL cells (**P**). Scale bars = 50 μm. *P < 0.05 by the Kruskal–Wallis test. *N* = 3 per group. Representative flow cytometric images showing death of 661W cone photoreceptor cells (**Q**) and the corresponding quantitative analysis of percentages of apoptotic (Annexin V^+^PI^−^plus Annexin V^+^PI^+^) 661W cells (**R**). The MSC group, Transwell co-culture of MNU-treated 661W cells with MSCs; the GW4869 group, GW4869 was used for MSC preconditioning before co-culture; the EXO group, MSC-derived exosomes were added to MNU-treated 661W cells. PI propidium iodide. *P < 0.05 by the Kruskal–Wallis test. *N* = 3 per group. Data represent median ± range for (**B**), (**I**), (**P**), and (**R**). Data are represented as box (25th, 50th, and 75th percentiles) and whisker (range) plots for (**D**), (**E**), (**G**), (**K**), (**L**), and (**N**).

Next, we investigated whether therapeutic effects of MSCT on MNU-provoked retinal damages were indeed mediated by paracrine mechanisms, particularly exosomes. GW4869, which blocks generation of exosomes [36, 37] as verified here (Fig. S1G), was applied for preconditioning of MSCs before transplantation. Notably, GW4869 pretreatment did not affect the biodistribution of injected MSCs in the retina (Fig. S1H), nor influence the viability or the differentiation of MSCs (Fig. S1I–K). Hematoxylin and eosin (H&E) staining images demonstrated that the application of GW4869 efficiently suppressed therapeutic effects of MSCT on MNU-induced retinal damages, resulted in failure of transplanted MSCs to preserve ONL thickness at 3 d (Fig. 2A, B). GW4869-dampened therapeutic efficacy of MSCT was also significant in both scotopic and photopic ERG analysis, in which MNU-triggered loss of retinal function was not rescued at 3 d (Fig. 2C–G). Similar phenomena were observed at 7 d after MNU injection, when transplantation of GW4869-pretreated MSCs demonstrated weaker protective effects compared to normal MSCs (Fig. S3).

To direct evaluate the effects of exosomes, we collected exosomes from cultured MSCs by serial centrifuges and ultracentrifuges, and verified their identity as EVs according to morphological characteristics and specific markers (Fig. S1D–F). Importantly, intravitreal transplantation of exosomes remarkably counteracted detrimental effects of MNU on the retina, leading to maintained ONL thickness and ERG function at 3 d despite MNU injection (Fig. 2H–N). The protective effects of EXOT against MNU injury were also prominent at 7 d (Fig. S2H–L) with remarkable preservance of cone photoreceptors and their function in the retina (Fig. S2M–O). Furthermore, to examine whether exosomes indeed regulate survival of photoreceptors, we analyzed TUNEL staining in retinal tissues in vivo and confirmed that GW4869 preconditioning prevented MSCT to alleviate MNU-induced photoreceptor apoptosis, while EXOT mimicked the protective effects of MSCT on photoreceptor viability (Fig. 2O, P). In vitro cellular apoptosis assay additionally proved that exosomal release determined beneficial effects of co-cultured MSCs on MNU-treated 661W cells (Fig. 2Q, R). Collectively, these findings indicated that exosomes mediates therapeutic effects of MSCT on MNU-provoked retinal photoreceptor damages.

### MSCT and EXOT exert long-term effects to protect photoreceptor and retina against degeneration

**Fig. 3:**
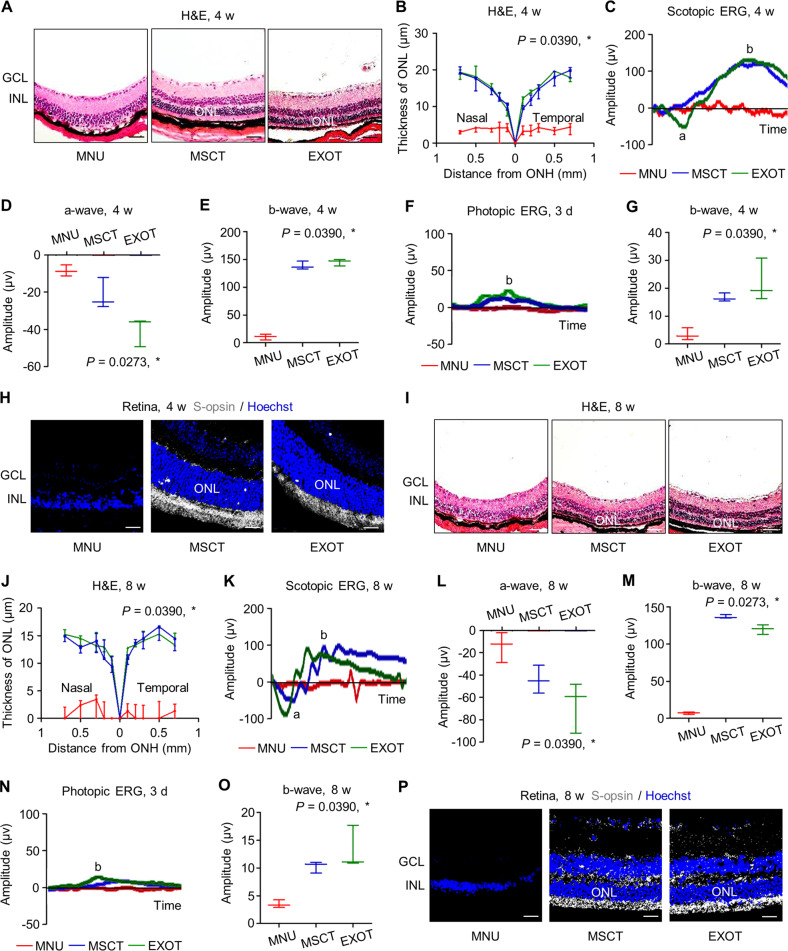
Intravitreal transplantation of mesenchymal stem cells (MSCs) and exosomes (EXO) maintains long-term photoreceptor viability against *N*-methyl-*N*-nitrosourea (MNU)-induced retinal degeneration. Representative hematoxylin and eosin (H&E) staining images of retinal tissues (**A**) and the corresponding quantitative analysis of ONL thickness (**B**). MSCT mesenchymal stem cell transplantation after MNU injection, EXOT exosomal transplantation after MNU injection, GCL ganglion cell layer, INL inner nuclear layer, ONL outer nuclear layer, ONH optic nerve head. Scale bars = 50 μm. *P < 0.05 by the Kruskal–Wallis test for area under curve (AUC). *N* = 3 per group. Representative scotopic electroretinography (ERG) waveforms (**C**) and the corresponding quantitative analyses of amplitude changes of a-wave (**D**) and b-wave (**E**). *P < 0.05 by the Kruskal–Wallis tests. *N* = 3 per group. Representative photopic ERG waveforms (**F**) and the corresponding quantitative analysis of b-wave amplitude changes (**G**). *P < 0.05 by the Kruskal–Wallis tests. *N* = 3 per group. **H** Representative immunofluorescent (IF) staining images of retinal tissues showing cone photoreceptor bodies (white) counterstained by Hoechst 33342 (blue). Scale bars = 25 μm. Representative H&E staining images of retinal tissues (**I**) and the corresponding quantitative analysis of ONL thickness (**J**). Scale bars = 50 μm. *P < 0.05 by the Kruskal–Wallis test for AUC. *N* = 3 per group. Representative scotopic ERG waveforms (**K**) and the corresponding quantitative analyses of amplitude changes of a-wave (**L**) and b-wave (**M**). *P < 0.05 by the Kruskal–Wallis tests. *N* = 3 per group. Representative photopic ERG waveforms (**N**) and the corresponding quantitative analysis of b-wave amplitude changes (**O**). *P < 0.05 by the Kruskal–Wallis tests. *N* = 3 per group. **P** Representative IF staining images of retinal tissues showing cone photoreceptor bodies (white) counterstained by Hoechst 33342 (blue). Scale bars = 25 μm. Data represent median ± range for (**B**), (**D**), (**E**), (**G**), (**J**), (**L**), (**M**), and (**O**).

We continued to investigate whether a single intravitreal injection of MSCs and exosomes could have a long-lasting protection of photoreceptors. We found that at 4 weeks in the experiment, MSCT and EXOT were still effective in preserving the ONL thickness and retinal scotopic ERG responses (Fig. 3A–E), with the protection of cone photoreceptor function in photopic ERG analysis (Fig. 3F, G) and S-opsin^+^ cone photoreceptor survival (Fig. 3H). Even at 8 weeks post to MNU challenge, MSCT and EXOT showed efficacy to protect the retina from degeneration (Fig. 3I–M), as also confirmed by preserved cone photoreceptors and their function (Fig. 3N–P). These results suggested long-term therapeutic effects of MSCT and EXOT despite only one-time injection.

**Fig. 4:**
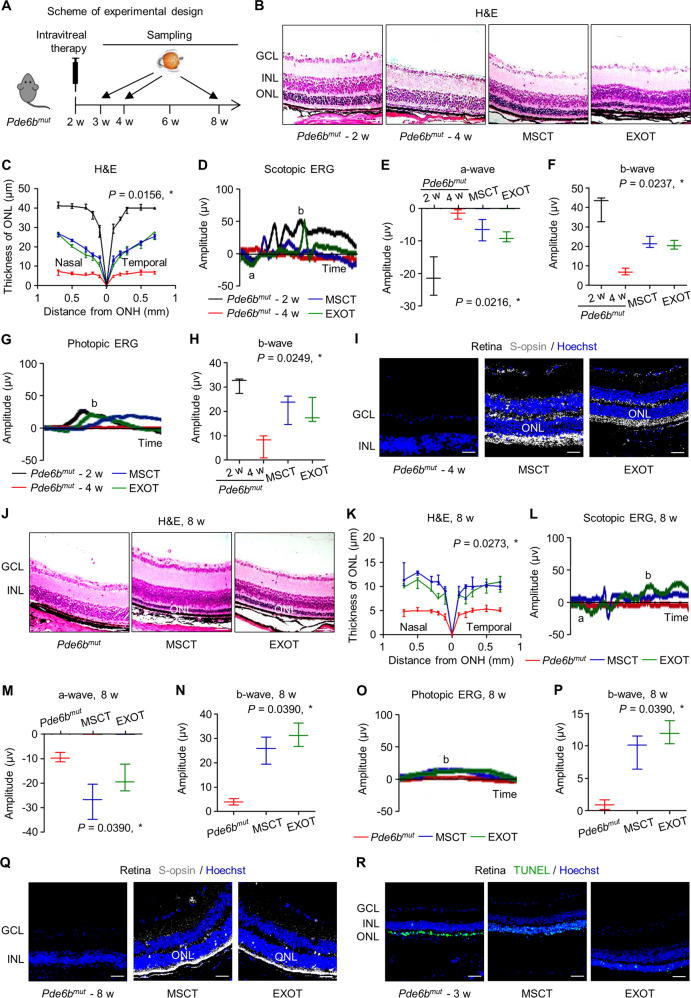
Intravitreal transplantation of mesenchymal stem cells (MSCs) and exosomes (EXO) ameliorates photoreceptor loss and retinal degeneration in the *Phosphodiesterase 6b* gene mutant (*Pde6b*^*mut*^) mouse model. **A** Schematic diagram demonstrating the study design of in vivo experiments on *Pde6b*^*mut*^ retinal degeneration. Representative hematoxylin and eosin (H&E) staining images of retinal tissues (**B**) and the corresponding quantitative analysis of ONL thickness (**C**). MSCT mesenchymal stem cell transplantation, EXOT exosomal transplantation, GCL ganglion cell layer, ONH optic nerve head. Scale bars = 50 μm. *P < 0.05 by the Kruskal–Wallis test for area under curve (AUC). *N* = 3 per group. Representative scotopic electroretinography (ERG) waveforms (**D**) and the corresponding quantitative analyses of amplitude changes of a-wave (**E**) and b-wave (**F**). *P < 0.05 by the Kruskal–Wallis tests. *N* = 3 per group. Representative photopic ERG waveforms (**G**) and the corresponding quantitative analysis of b-wave amplitude changes (**H**). *P < 0.05 by the Kruskal–Wallis tests. *N* = 3 per group. **I** Representative immunofluorescent (IF) staining images of retinal tissues showing cone photoreceptor bodies (white) counterstained by Hoechst 33342 (blue). Scale bars = 25 μm. Representative H&E staining images of retinal tissues (**J**) and the corresponding quantitative analysis of ONL thickness (**K**). Scale bars = 50 μm. *P < 0.05 by the Kruskal–Wallis test for AUC. *N* = 3 per group. Representative scotopic ERG waveforms (**L**) and the corresponding quantitative analyses of amplitude changes of a-wave (**M**) and b-wave (**N**). *P < 0.05 by the Kruskal–Wallis tests. *N* = 3 per group. Representative photopic ERG waveforms (**O**) and the corresponding quantitative analysis of b-wave amplitude changes (**P**). *P < 0.05 by the Kruskal–Wallis tests. *N* = 3 per group. **Q** Representative immunofluorescent (IF) staining images of retinal tissues showing cone photoreceptor bodies (white) counterstained by Hoechst 33342 (blue). Scale bars = 25 μm. **R** Representative terminal deoxynucleotidyl transferase dUTP nick end labeling (TUNEL, green) staining images of retinal tissues counterstained by Hoechst 33342 (blue). Scale bars = 50 μm. Data represent median ± range for (**C**), (**E**), (**F**), (**H**), (**K**), (**M**), (**N**), and (**P**).

We further applied a genetic mouse retinal degeneration model caused by a nonsense mutation of *Phosphodiesterase 6b* gene (*Pde6b*^*mut*^), as previously established to mimick RP [38], and treated the mice with MSCs and exosomes at 2-week old (Fig. 4A). We discovered that the *Pde6b*^*mut*^ mice at 4 weeks of age exhibited fast retinal degeneration, with substantial loss of ONL thickness (Fig. 4B, C), electrophysiological function (Fig. 4D–H), and cone photoreceptors (Fig. 4I). Expectedly, MSCT and EXOT prevented the above degeneration (Fig. 4B–I). Importantly, MSCT and EXOT significantly alleviated alterations of retinal morphology, loss of electrophysiological function, and diminished cone photoreceptors of *Pde6b*^*mut*^ mice at 8 weeks of age (Fig. 4J–Q). These effects can be attributed to MSCT and EXOT rescue of photoreceptor apoptosis at the early stage of *Pde6b*^*mut*^ mice at 3-week old (Fig. 4R). Collectively, these findings indicated long-term therapeutic effects of intravitreal MSCs and exosomes in a natural occurring retinal degeneration model.

### miR-21 deficiency aggravates MNU-driven retinal injury and can be restrained by EXOT

**Fig. 5:**
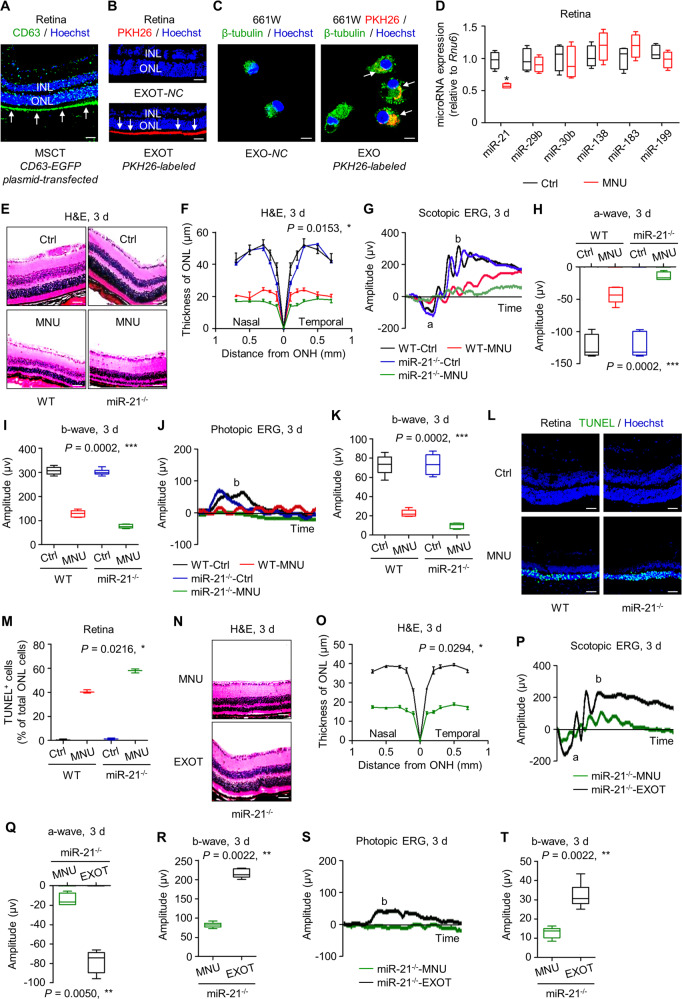
miR-21 deficiency aggravates *N*-methyl-*N*-nitrosourea (MNU)-induced photoreceptor apoptosis and retinal degeneration, which is restrained by transplantation of mesenchymal stem cell (MSC)-derived exosomes. **A** Tracing of CD63-EGFP plasmid-transfected MSCs (green, CD63) in the retina tissue counterstained by Hoechst 33342 (blue) after intravitreal injection for 24 h. MSCT mesenchymal stem cell transplantation, INL inner nuclear layer, ONL outer nuclear layer. Scale bar = 50 μm. **B** Tracing of PKH26-labeled exosomes (red) in the retina tissue counterstained by Hoechst 33342 (blue) after intravitreal injection for 24 h. EXOT exosomal transplantation, NC negative control, injection of EXO without staining. Scale bar = 50 μm. **C** Tracing of PKH26-labeled, MSC-derived exosomes (red) in 661W cone photoreceptor cells during in vitro treatment. 661W cells are demonstrated by β-tubulin immunostaining for microtubules (green), counterstained by Hoechst 33342 (blue). NC negative control, treatment of EXO without staining. Scale bar = 10 μm. **D** Quantitative real-time polymerase chain reaction (qRT-PCR) analysis of expression levels of multiple microRNAs in retina tissues, normalized to *Rnu6*. Ctrl control. *P < 0.05 by the Mann–Whitney *U* test. *N* = 4 per group. Representative hematoxylin and eosin (H&E) staining images of retinal tissues (**E**) and the corresponding quantitative analysis of outer nuclear layer (ONL) thickness (**F**). WT wild-type mice, miR-21^−/−^ mice deficient for miR-21, ONH optic nerve head. Scale bars = 50 μm. *P < 0.05 by the Kruskal–Wallis test for area under curve (AUC). *N* = 3 per group. Representative scotopic electroretinography (ERG) waveforms (**G**) and the corresponding quantitative analyses of amplitude changes of a-wave (**H**) and b-wave (**I**). *P < 0.05 by the Kruskal–Wallis tests. *N* = 6 per group. Representative photopic ERG waveforms (**J**) and the corresponding quantitative analysis of b-wave amplitude changes (**K**). *P < 0.05 by the Kruskal–Wallis tests. *N* = 6 per group. Representative terminal deoxynucleotidyl transferase dUTP nick end labeling (TUNEL, green) staining images of retinal tissues counterstained by Hoechst 33342 (blue) (**L**) and the corresponding quantitative analysis of percentages of TUNEL^+^ cells over total ONL cells (**M**). Scale bars = 50 μm. *P < 0.05 by the Kruskal–Wallis test. *N* = 3 per group. Representative H&E staining images of retinal tissues (**N**) and the corresponding quantitative analysis of ONL thickness (**O**). miR-21-deficient mice were treated by MNU with or without EXOT. Scale bars = 50 μm. *P < 0.05 by the Mann–Whitney *U* test for AUC. *N* = 3 per group. Representative scotopic ERG waveforms recorded for retinal functional analysis (**P**) and the corresponding quantitative analyses of amplitude changes of a-wave (**Q**) and b-wave (**R**). *P < 0.05 by the Mann–Whitney *U* tests. *N* = 6 per group. Representative photopic ERG waveforms (**S**) and the corresponding quantitative analysis of b-wave amplitude changes (**T**). *P < 0.05 by the Kruskal–Wallis tests. *N* = 6 per group. Data represent median ± range for (**F**), (**M**), and (**O**). Data are represented as box (25th, 50th, and 75th percentiles) and whisker (range) plots for (**D**), (**H**), (**I**), (**K**), (**Q**), (**R**), and (**T**).

In the next experiments, we aimed to decipher how MSC-derived exosomes maintained retinal photoreceptor integrity. Using CD63-EGFP plasmid-transfected MSCs, we firstly confirmed that injected MSCs were able to release exosomes in vivo to the ONL (Fig. 5A). Direct tracing of PKH26-labeled exosomes further verified that after intravitreal transplantation, the exosomes were concentrated and linear along the posterior edge of the ONL, suggesting specific targeting of photoreceptors gathering around photoreceptor nuclei (Fig. 5B). In vitro tracing confirmed that 661W photoreceptors were capable of uptaking MSC-derived exosomes, suggesting exosome-mediated cellular communications (Fig. 5C).

The long-term effects of MSCT and EXOT on the retina indicated epigenetic therapeutic mechanisms, among which microRNA regulation may exist [26]. It has also been well documented that exosomes transfer microRNAs for the regulation of recipient cell function in therapeutics [24]. To figure out the potential microRNAs mediating therapeutic effects of exosomes in this study, we took a look on the expression changes of several retina-regulating microRNAs upon the phototoxin MNU treatment [29, 39–41]. Quantitative real-time polymerase chain reaction (qRT-PCR) analysis showed that of the selected microRNAs, only miR-21 expression was downregulated by MNU in the retina (Fig. 5D). To investigate whether decline of miR-21 expression contributed to the pathogenesis of retinal degeneration, we applied mice deficient for miR-21 (miR-21^−/−^) [42, 43] and challenged them with MNU. As expected, MNU induced more prominent retinal damages in miR-21^−/−^ mice compared to wild-type (WT) mice at 3 d, leading to more loss of ONL thickness and worse electrophysiological function of the retina (Fig. 5E–K). Interestingly, miR-21^−/−^ mice per se did not show retinal deficiency, indicating that effects of miR-21 were in responses to cell death stimuli (Fig. 5E–K). TUNEL staining further demonstrated that miR-21^−/−^ mice after MNU injection developed higher apoptotic rate of the photoreceptors compared to WT mice with MNU treatment (Fig. 5L, M). Importantly, MNU-induced retinal damages in miR-21^−/−^ mice were also rescued by EXOT, exhibiting improved ONL thickness and retinal function not only at 3 d (Fig. 5N–T) but also at 7 d (Fig. S4A–G). Notably, EXOT also helped photoreceptors to survive after MNU challenge in miR-21^−/−^ mice (Fig. S4H, I). These results suggested that miR-21 deficiency aggravates MNU-driven retinal injury and can be restrained by EXOT.

### miR-21 protects photoreceptors against MNU-triggered apoptosis and can be transferred from MSCs for functional regulation

**Fig. 6:**
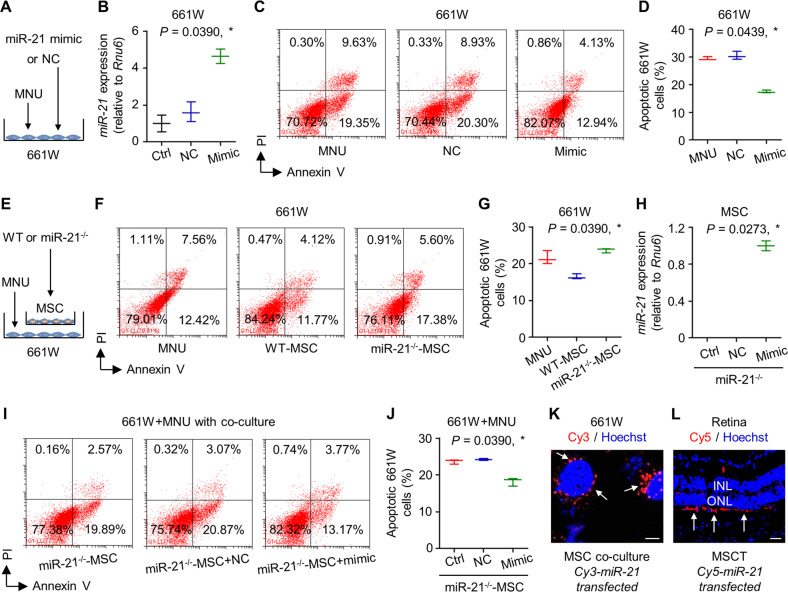
miR-21 protects 661W cone photoreceptor cells against *N*-methyl-*N*-nitrosourea (MNU)-induced apoptosis and can be transferred from mesenchymal stem cells (MSCs). **A** Diagram demonstrating the experimental design for analyzing direct effects of miR-21 on 661W cells. NC negative control of miR-21 mimics. **B** Quantitative real-time polymerase chain reaction (qRT-PCR) analysis of expression levels of *miR-21* in 661W cells, normalized to *Rnu6*. Ctrl control. *P < 0.05 by the Kruskal–Wallis test. *N* = 3 per group. Representative flow cytometric images showing death of 661W cone photoreceptor cells (**C**) and the corresponding quantitative analysis of percentages of apoptotic (Annexin V^+^PI^−^ plus Annexin V^+^PI^+^) 661W cells (**D**). 661W cells were treated with MNU with or without transfection of NC or miR-21 mimics. PI propidium iodide. *P < 0.05 by the Kruskal–Wallis test. *N* = 3 per group. **E** Diagram demonstrating the experimental design for analyzing indirect effects of MSCs on 661W cells. WT wild-type mice, miR-21^−/−^, mice deficient for miR-21. Representative flow cytometric images showing death of 661W cone photoreceptor cells (**F**) and the corresponding quantitative analysis of percentages of apoptotic (Annexin V^+^PI^−^ plus Annexin V^+^PI^+^) 661W cells (**G**). MNU-treated 661W cells were co-cultured in the Transwell system with or without different MSCs. *P < 0.05 by the Kruskal–Wallis test. *N* = 3 per group. **H** qRT-PCR analysis of expression levels of *miR-21* in MSCs, normalized to *Rnu6*. *P < 0.05 by the Kruskal–Wallis test. *N* = 3 per group. Representative flow cytometric images showing death of 661W cone photoreceptor cells (**I**) and the corresponding quantitative analysis of percentages of apoptotic (Annexin V^+^PI^−^ plus Annexin V^+^PI^+^) 661W cells (**J**). 661W cells were treated with MNU in the Transwell co-culture system with MSCs, which were transfected with or without NC or miR-21 mimics. *P < 0.05 by the Kruskal–Wallis test. *N* = 3 per group. **K** Tracing of Cy3-labeled, MSC-derived miR-21 (red) in 661W cells during co-culture with MSCs, counterstained by Hoechst 33342 (blue). Scale bar = 10 μm. **L** Tracing of Cy5-labeled, MSC-derived miR-21 (red) in the retina tissue counterstained by Hoechst 33342 (blue) after intravitreal injection of MSCs for 24 h. MSCT mesenchymal stem cell transplantation, INL inner nuclear layer, ONL outer nuclear layer. Scale bar = 50 μm. Data represent median ± range for (**B**), (**D**), (**G**), (**H**), and (**J**).

Next, we intended to verify miR-21 as a crucial regulator of photoreceptor survival before evaluating its function to mediate therapeutic effects of EXOT. As depicted, direct transfection of miR-21 mimics and its negative control (NC) into cultured 661W cells was applied (Fig. 6A), and the efficacy of transfection was confirmed by qRT-PCR (Fig. 6B). Flow cytometric analysis demonstrated that transfection of miR-21 mimics significantly protected 661W cells from MNU-induced apoptosis (Fig. 6C, D). We then further evaluated whether miR-21 mediated regulation of MSCs on photoreceptor survival. Using a Transwell co-culture system, we found that MSCs indeed counteracted MNU-induced 661W cell apoptosis via paracrine effects, while MSCs deficient for miR-21 were limited in the paracrine protection (Fig. 6E–G). To confirm that miR-21 per se mediated effects of MSCs in co-culture, we introduced miR-21 mimics into the miR-21^−/−^ mouse MSCs, and confirmed their efficacy in safeguarding MSC protection on MNU-induced 661W cell apoptosis (Fig. 6H–J). To examine that whether miR-21 can be transferred from MSCs to photoreceptors, we transfected MSCs with Cy3-labeled miR-21 mimics before co-culture, and detected the Cy3 fluorescent signal surrounding the nuclei of co-cultured 661W cells (Fig. 6K). Further in vivo tracing analysis after injection of Cy5-labeled miR-21-transfected MSCs confirmed that miR-21 can be transferred to photoreceptors in the retina (Fig. 6L). These data collectively suggested that miR-21 protects photoreceptors against MNU-triggered apoptosis and can be transferred from MSCs for functional regulation.

### Exosomal counteraction of MNU damages on photoreceptors involves inhibition of the miR-21 target Pdcd4

**Fig. 7:**
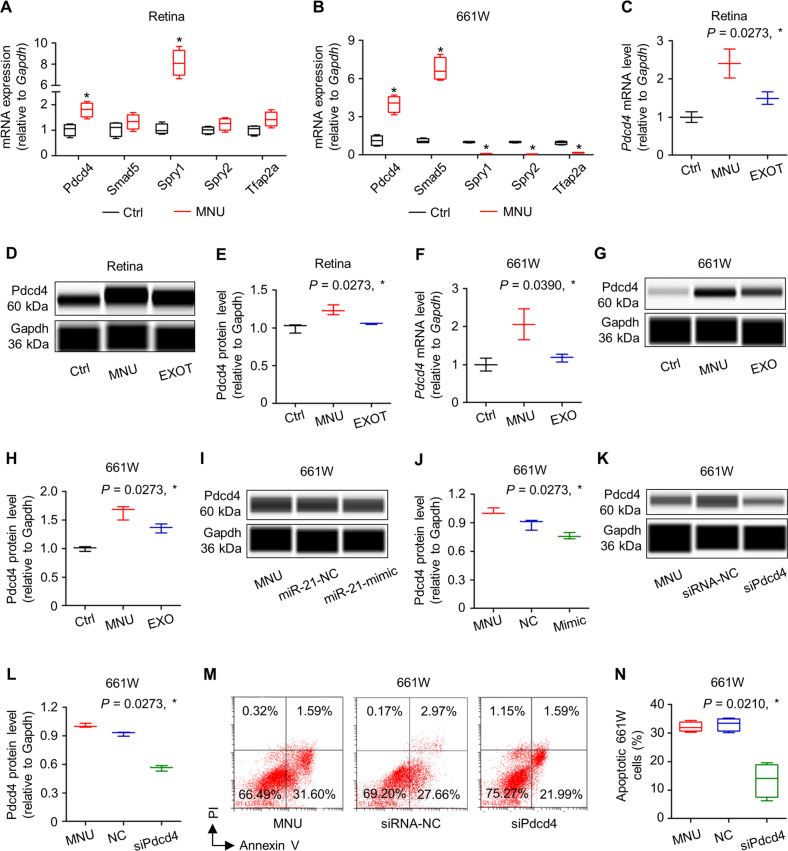
Exosomal counteraction of *N*-methyl-*N*-nitrosourea (MNU) damages on photoreceptor cells involves inhibition of the miR-21 target programmed cell death 4 (Pdcd4). Quantitative real-time polymerase chain reaction (qRT-PCR) analysis of mRNA expression levels of multiple targets of miR-21 in retina tissues (**A**) and 661W cone photoreceptor cells (**B**), normalized to *Glyceraldehyde-3-phosphate dehydrogenase* (*Gapdh*). Ctrl control, *Spry1* *Sprouty 1*, *Spry 2* *Sprouty 2*, *Tfap2a* *Transcription factor AP-2-alpha*. *P < 0.05 by the Mann–Whitney *U* tests. *N* = 4 per group. qRT-PCR analysis of mRNA expression levels (**C**) and western blot analysis of protein expression levels (D) with corresponding quantification (**E**) of Pdcd4 in retina tissues, normalized to Gapdh. EXOT exosomal transplantation after MNU injection. *P < 0.05 by the Kruskal–Wallis tests. *N* = 3 per group. qRT-PCR analysis of mRNA expression levels (**F**) and western blot analysis of protein expression levels (**G**) with corresponding quantification (**H**) of Pdcd4 in 661W cells, normalized to Gapdh. *P < 0.05 by the Kruskal–Wallis tests. *N* = 3 per group. Western blot analysis of protein expression levels (**I**) with corresponding quantification (**J**) of Pdcd4 in 661W cells, normalized to Gapdh. NC negative control of miR-21 mimics. 661W cells were treated with MNU with or without transfection of NC or miR-21 mimics. *P < 0.05 by the Kruskal–Wallis test. *N* = 3 per group. Western blot analysis of protein expression levels (**K**) with corresponding quantification (**L**) of Pdcd4 in 661W cells, normalized to Gapdh. siRNA-NC negative control of small interfering RNA for Pdcd4, siPdcd4 small interfering RNA for Pdcd4. 661W cells were treated with MNU with or without transfection of siRNA-NC or siPdcd4. *P < 0.05 by the Kruskal–Wallis test. *N* = 3 per group. Representative flow cytometric images showing death of 661W cone photoreceptor cells (**M**) and the corresponding quantitative analysis of percentages of apoptotic (Annexin V^+^PI^−^ plus Annexin V^+^PI^+^) 661W cells (**N**). 661W cells were treated with MNU with or without transfection of siRNA-NC or siPdcd4. *P < 0.05 by the Kruskal–Wallis test. *N* = 4 per group. Data represent median ± range for (**C**), (**E**), (**F**), (**H**), (**J**), and (**L**). Data are represented as box (25th, 50th, and 75th percentiles) and whisker (range) plots for (**A**), (**B**), and (**N**).

We then investigated the functional target of miR-21 in regulating photoreceptor apoptosis. qRT-PCR screening of reported miR-21 targets [44–47] in both the retinal tissues and 661W cone photoreceptors demonstrated that the mRNA expression levels of Pdcd4, which is a valid target of miR-21 and has been reported for neuroprotection [48], were consistently upregulated by MNU treatments, correlated with downregulated miR-21 expression (Fig. 7A, B). Further evaluation showed that not only in the mRNA levels but also in the protein levels, Pdcd4 expression was induced by MNU but suppressed by EXOT in the retina, suggesting pathological and therapeutic responses (Figs. 7C–E and S5A). Examination of Pdcd4 expression in 661W cells confirmed that EXOT restored MNU-triggered upregulation of Pdcd4 (Figs. 7F–H and S5B). To verify that Pdcd4 expression was indeed regulated by miR-21, we analyzed 661W cells transfected with miR-21 mimics, and found that miR-21 mimics repressed Pdcd4 expression at the protein level (Figs. 7I, J and S5C). To prove that Pdcd4 was functionally important for photoreceptor survival, we transfected 661W cells with a small interfering RNA (siRNA) for Pdcd4 (siPdcd4) and challenged them with MNU (Figs. 7K, L and S5D). Flow cytometric analysis demonstrated that transfection of siPdcd4, but not its NC, effectively protected 661W cells against MNU-induced apoptosis (Fig. 7M, N). These data suggested that exosomal counteraction of MNU damages on photoreceptors involves inhibition of the miR-21 target Pdcd4.

### Exosomal miR-21 serves as an effective therapeutic for MNU-induced photoreceptor apoptosis and retinal degeneration

**Fig. 8:**
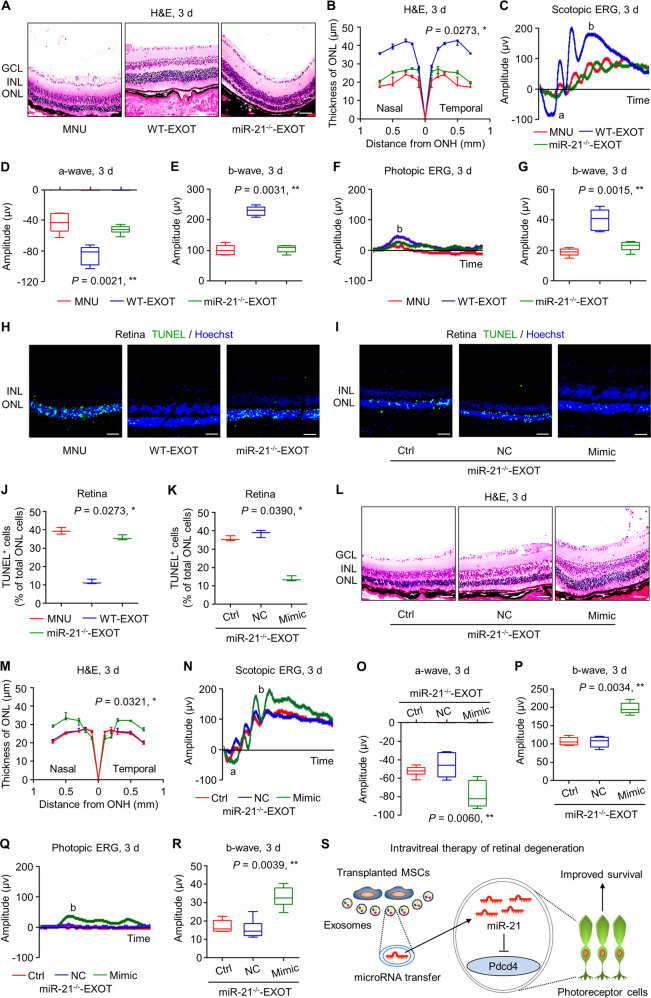
Exosomal miR-21 counteracts *N*-methyl-*N*-nitrosourea (MNU)-induced photoreceptor apoptosis and retinal degeneration. Representative hematoxylin and eosin (H&E) staining images of retinal tissues (**A**) and the corresponding quantitative analysis of outer nuclear layer (ONL) thickness (**B**). WT-EXOT transplantation of exosomes derived from wild-type mesenchymal stem cells (MSCs) after MNU injection, miR-21^−/−^-EXOT transplantation of exosomes derived from miR-21-deficient MSCs after MNU injection, GCL ganglion cell layer, INL inner nuclear layer, ONH optic nerve head. Scale bars = 50 μm. *P < 0.05 by the Kruskal–Wallis test for area under curve (AUC). *N* = 3 per group. Representative scotopic electroretinography (ERG) waveforms (**C**) and the corresponding quantitative analyses of amplitude changes of a-wave (**D**) and b-wave (**E**). *P < 0.05 by the Kruskal–Wallis tests. *N* = 6 per group. Representative photopic ERG waveforms (**F**) and the corresponding quantitative analysis of b-wave amplitude changes (**G**). *P < 0.05 by the Kruskal–Wallis tests. *N* = 6 per group. Representative terminal deoxynucleotidyl transferase dUTP nick end labeling (TUNEL, green) staining images of retinal tissues counterstained by Hoechst 33342 (blue) (**H**) and the corresponding quantitative analysis of percentages of TUNEL^+^ cells over total ONL cells (**J**). Scale bars = 50 μm. *P < 0.05 by the Kruskal–Wallis test. *N* = 3 per group. Representative TUNEL (green) staining images of retinal tissues counterstained by Hoechst 33342 (blue) (**I**) and the corresponding quantitative analysis of percentages of TUNEL^+^ cells over total ONL cells (**K**). Ctrl control, NC negative control of miR-21 mimics. MNU-injected mice were transplanted with exosomes derived from miR-21-deficient MSCs, which were transfected with or without NC or miR-21 mimics. Scale bars = 50 μm. *P < 0.05 by the Kruskal–Wallis test. *N* = 3 per group. Representative H&E staining images of retinal tissues (**L**) and the corresponding quantitative analysis of ONL thickness (**M**). Scale bars = 50 μm. *P < 0.05 by the Kruskal–Wallis test for AUC. *N* = 3 per group. Representative scotopic ERG waveforms (**N**) and the corresponding quantitative analyses of amplitude changes of a-wave (**O**) and b-wave (**P**). *P < 0.05 by the Kruskal–Wallis tests. *N* = 6 per group. Representative photopic ERG waveforms (**Q**) and the corresponding quantitative analysis of b-wave amplitude changes (**R**). *P < 0.05 by the Kruskal–Wallis tests. *N* = 6 per group. **S** Diagram showing the synopsis of the findings. Data represent median ± range for (**B**), (**J**), (**K**), and (**M**). Data are represented as box (25th, 50th, and 75th percentiles) and whisker (range) plots for (**D**), (**E**), (**G**), (**O**), (**P**), and (**R**).

The above results prompted us to decipher whether miR-21 mediated therapeutic effects of EXOT on retinal degeneration. By using MSCs derived from WT and miR-21^−/−^ mice, we identified that MSCs from WT mice secreted miR-21-carrying EXO, while EXO released by MSCs from miR-21^−/−^ mice failed to show miR-21 expression (Fig. S1L). We discovered that donor miR-21 deficiency remarkably diminished effects of EXOT to preserve morphological and functional integrity of the retina, leaving the ONL thickness and both scotopic and photopic ERG amplitudes unrecovered at 3 d (Fig. 8A–G) and 7 d (Fig. S6A–G). TUNEL analysis confirmed that miR-21^−/−^ EXOT was less efficient to protect photoreceptors against MNU-induced apoptosis, whereas after transfection of miR-21 mimics into miR-21^−/−^ MSCs, EXOT regained the efficacy to improve photoreceptor survival after MNU treatment (Fig. 8H–K). Furthermore, miR-21 mimics significantly rescued miR-21^−/−^ EXOT to overcome the failure in counteracting MNU-induced morphological and electrophysiological damages of the retina (Figs. 8L–R and S6H–N). A diagram showing the synopsis of our findings is provided (Fig. 8S). Taken together, these data indicated that exosomal miR-21 serves as an effective therapeutic for MNU-induced photoreceptor apoptosis and retinal degeneration.

## Discussion

Photoreceptor apoptosis represents one key pathogenesis of retinal degeneration and one of the ultimate reasons of visual loss with only few available treatments [1, 2]. Whether and how MSCT protects photoreceptors against apoptotic injuries remains unknown. Here, we discovered that intravitreal MSCT long-lastingly counteracted photoreceptor apoptosis and alleviates retinal degeneration, which was exerted through exosomes for intercellular communication between injected MSCs and photoreceptors. We further identified that miR-21, which critically maintained photoreceptor viability by targeting Pdcd4, was transferred from MSCs and mediate therapeutic effects of EXOT. Our findings uncover the efficacy and mechanism of MSCT-based photoreceptor protection, which also indicates exosomal miR-21 as a therapeutic for retinal degeneration.

Degenerative retinal diseases are significant causes of incurable sight loss but the therapeutic options are currently limited [49]. Despite their very different and multifactorial etiologies, the various retinal diseases lead to the same end-result for visual impairment, namely the loss of photoreceptors [1, 2]. Nevertheless, photoreceptors fail to regenerate upon injuries, which therefore demands cell replacement or rescue technologies [50]. In this regard, transplantation of embryonic stem cell-differentiated RPE to support photoreceptors has obtained encouraging results to alleviate retinal degeneration from phase I/II trials [51]. For photoreceptors per se, however, although that the apoptotic pathogenesis have been studied, targeting photoreceptor apoptosis or replenishing photoreceptors as potential approaches to retinal degeneration are still in developing [1, 2]. Extensively efficient to ameliorate retinal traumatic, ischemic, and oxidative injuries [7, 18] as well as retarding inflammatory and diabetic retinopathies [8, 52], MSCT represents promising cell-based therapeutics which also rescues photoreceptor deficiency in mice with genetic defects [9–11]. Notably, there are studies reporting retinal damages and ocular complications induced by intravitreal or subretinal MSCT, indicating that stem cell delivery should still be applied with caution [53, 54]. In this study, we for the first time revealed that MSCT protected photoreceptors against specific apoptotic stimulus in vivo, and further identified EXOT as cell-free alternative strategy to overcome potential drawbacks of cell transplantation with the beneficial effects preserved. Moreover, we discovered that a single intravitreal injection of MSCs or exosomes has a long-lasting protection of photoreceptors in both the MNU pharmacological model and the *Pde6b*^*mut*^ genetic model with ameliorated loss of either scotopic (mixed rod and cone responses) or photopic (cone-mediated) ERG responses, which therefore provides promise for establishing novel therapeutics of retinal degeneration.

It is recognized that MSCT maintains tissue homeostasis through either paracrine effects to establish beneficial microenvironments or through inhabitation in recipient tissues to replenish deficient cells [14, 55]. After intravitreal transplantation, exogenous MSCs have been traced to remain in the vitreous body, in which no retinal incorporation has been reported [56–58]. Nevertheless, it has been claimed in MSCT treating a mouse model of RP that transplanted MSCs morphologically integrated into the RPE, while not being detected in other retinal layers [10]. Additionally, in vitro experiments indicated regulation of MSCs by the retinal microenvironment, in that RPE cell-conditioned medium and photoreceptor outer segments stimulate differentiation of MSCs toward the RPE cell phenotype, but the in vivo functional evidence of MSC replacing retinal cells is lacking [59]. Here, we showed that the biodistribution of injected MSCs was primarily at the ONL, indicating close functional correlationships with recipient photoreceptors and potential needs of damaging photoreceptors for recovery. However, MSCs were able to release exosomes for therapeutic effects at the ONL, and effects but not biodistribution of MSCs were diminished upon blockade of exosomal generation. Therefore, it can be expected that MSCT improves retinal integrity and function via paracrine effects, as also confirmed by the identification of various neuroprotective and anti-inflammatory factors secreted, such as nerve growth factor, basic fibroblast growth factor, and tumor necrosis factor alpha-stimulated gene-6 [8, 16]. It has additionally been reported that MSC-derived EVs enhance functional recovery of retina after ischemia-reperfusion damages and optic nerve crush injuries, and that transplanted EVs can be uptaken by RGCs and microglia [17, 18]. In this study, we further revealed that EXOT efficiently and continuously counteracted phototoxin-induced and *Pde6b*^*mut*^ retinal degeneration by targeting photoreceptors, adding another dimensional to the current knowledge of paracrine-based MSC therapy of retinal disorders. Whether the injected MSCs can also proliferate and differentiate toward photoreceptors in vivo remain to be investigated in future studies.

Shuttle of microRNAs by EVs have been widely proved as significant contributors to tissue homeostasis and feasible therapeutics for diseases [23–25]. In the ocular system, it has been reported that EV-mediated microRNA communication may influence posttranscriptional regulation of retinal development, and that microRNAs carried by circulating EVs can be used as prognostic biomarkers for retinopathy [20, 21]. microRNA-dependent mechanisms have also been implicated in MSC-derived EVs to treat corneal fibrosis and optic nerve injury [17, 60]. In this study, we first identified an individual microRNA, miR-21, as a contributor to photoreceptor viability in vivo and a mediator of therapeutic effects of EXOT on retinal dysfunction. Notably, miR-21 has been previously documented mainly as a negative regulator of retinal health, such as inhibition of neovascularization in the ischemic retina by targeting tissue inhibitor of metalloproteinase 3 [61], induction of Müller cell gliosis after optic nerve crush by regulating glial fibrillary acidic protein [39], promotion of autoimmune uveoretinitis by targeting interleukin-10 [62], and facilitation of the progression of retinoblastoma by targeting phosphatase and tensin homolog [63]. Besides, previous studies have shown that miR-21 can induce cell apoptosis by targeting S-phase kinase-associated protein 2 and B-cell lymphoma-2 [64, 65]. Our results showing miR-21 protection against retinal disorders only join with another study demonstrating that the miR-21/Pdcd4 axis regulates MSC-induced neuroprotection in glaucoma, indicating target-specific regulatory effects based on Pdcd4 inhibition [48]. Actually, miR-21 inhibition of Pdcd4 expression for antiapoptotic effects has additionally been reported in other systems [66, 67]. The detailed function and mechanisms of Pdcd4 in regulating photoreceptors and retinal degeneration in vivo remains to be elucidated in future studies.

## Materials and methods

### Animals

Animal experiments were performed following the Guidelines of Intramural Animal Use and Care Committees of Fourth Military Medical University, Xi’an Jiaotong University and the ARRIVE guidelines. Twelve-week WT C57BL/6 mice and miR-21^−/−^ mice (C57BL/6 background) (weight, 20–22 g; three male or female mice per cage) were purchased from the Jackson Laboratory, as we have used in previous studies [42, 43]. Two-week-old male or female *Pde6b*^*mut*^ mice with a nonsense mutation of *Pde6b* gene on a C57BL/6J background that result in the failure of protein production [38] were obtained from TC at Department of Clinical Medicine, Fourth Military Medical University. Mice were maintained with good ventilation and a 12-h light/dark cycle, and were kept feeding and drinking ad libitum.

### Cell culture

Isolation and culture of MSCs from mouse bone marrow were as previously described [68]. Briefly, whole bone marrow cells were seeded, incubated overnight, and rinsed with phosphate buffer saline (PBS) to remove the non-adherent cells. The adherent cells were cultured with alpha-Minimum Essential Medium supplemented with 20% fetal bovine serum (FBS), 2-mM l-glutamine, 100-U/ml penicillin, and 100-g/ml streptomycin (all from Invitrogen, USA) at 37 °C in a humidified atmosphere of 5% CO_2_. Primary MSCs were digested with 0.25% trypsin (MP Biomedicals, USA) and passaged for the following experiments after seeding at appropriate densities.

MSCs were verified according to the current standard [32, 33]. For flow cytometric analysis of the surface markers, MSCs at the first passage were collected and suspended in PBS supplemented with 3% FBS at 1 × 10^6^ cells/ml. MSCs were added with FITC-conjugated anti-mouse CD11b antibody (11-0112-82; eBioscience, USA), PE-conjugated anti-mouse CD29 antibody (12-0299-42; eBioscience, USA), FITC-conjugated anti-mouse CD34 antibody (11-0341-82; eBioscience, USA), PE-conjugated anti-mouse CD45 antibody (12-0451-82; eBioscience, USA), PE-conjugated anti-mouse CD31 antibody (12-0311-82; eBioscience, USA), and FITC-conjugated anti-mouse Stem cell antigen 1 (Sca1) antibody (11-5981-82; eBioscience, USA) at concentrations of 1:100. Nonimmune immunoglobulin of the same isotype was used as the NC. MSCs were incubated in 4 °C for 30 min in dark, and then washed twice with PBS supplemented with 3% FBS. The percentages of positively stained cells were determined with a flow cytometer (FACSAria; BD Biosciences, USA) equipped with the FACSDiva Version 6.1.3 software. For cell viability examinations, the seeded MSCs at the first passage were incubated with 20-μl 5-mg/ml methyl thiazolyl tetrazolium (MP Biomedicals, USA) for 4 h [55, 69]. The precipitates were extracted with 180-μl dimethyl sulfoxide (DMSO) and the absorbance was measured at the optical density of 490 nm. Cell viability fold changes were calculated accordingly. For osteogenic differentiation, the seeded MSCs at the first passage were induced in media containing 100-μg/ml ascorbic acid (MP Biomedicals, USA), 2-mM β-glycerophosphate (Sigma-Aldrich, USA) and 10-nM dexamethasone (Sigma-Aldrich, USA) for 14 d, and Alizarin red (Sigma-Aldrich, USA) staining was performed to determine the mineralization. For adipogenic differentiation, the seeded MSCs at the first passage were induced in media containing 0.5-mM isobutylmethylxanthine (MP Biomedicals, USA), 0.5-mM dexamethasone and 60-mM indomethacin (MP Biomedicals, USA) for 14 d, and Oil red O (Sigma-Aldrich, USA) staining was performed to determine the lipid droplet formation. Photographs were taken using an inverted optical microscope (CKX41; Olympus, Japan).

Culture of the 661W cell line was according to our previous study and verified for identity and non-contamination [70]. The 661W cell line was derived from mouse retinal tumors and has been characterized previously to be of cone photoreceptor cell lineage [71]. The 661W cell line was cultured in Dulbecco’s Modified Eagle Medium supplemented with 10% FBS, 2-mM l-glutamine, 100-U/ml penicillin, and 100-g/ml streptomycin (all from Invitrogen, USA) at 37 °C in a humidified atmosphere of 5% CO_2_. For co-culture of MSCs with 661W cells, 661W cells were seeded in 12-well plates as the bottom of a Transwell system (0.4-μm pore size; Corning, USA), while MSCs at the first passage were added into the upper chamber of the Transwell system for 48 h.

### Chemical treatments

MNU-provoked retinal degeneration model was established accordingly [70, 72] by intraperitoneal injection of MNU (Sigma-Aldrich, USA) at 40 mg/kg. MNU was freshly dissolved in sterile saline. Mice were sacrificed at indicated time points according to study design. In vitro treatment of 661W cells with MNU was performed at a concentration of 200 μg/ml for 6 h. For blockade of MSC generation of exosomes [36, 37], 10-μM GW4869 (Sigma-Aldrich, USA) was applied in MSC culture for 24 h, which was initially dissolved in DMSO into a stock solution of 5 mM and was diluted in culture media. The effects of GW4869 on MSC viability and osteogenesis were determined after wash-out procedures.

### Collection and identification of exosomes

Collection of MSC-derived exosomes was performed as stated before [73]. Briefly, MSCs at nearly confluence of the first passage were cultured in exosome-depleted media (complete media supernatant after overnight centrifugation at 100,000 *g*) for 48 h. Exosomes from supernatants were then isolated by the differential centrifugation protocol at 4 °C: 300 *g* for 10 min, 3000 *g* for 10 min, 10,000 *g* for 20 min, 100,000 *g* for 70 min, followed by washing with PBS for another centrifugation at 100,000 *g* for 70 min. Quantification of exosomes were performed by determining the concentration of total proteins using the Pierce BCA Protein Assay (Thermo Fisher Scientific, USA).

Exosomes were identified according to the criteria of EVs [74]. The number and size distribution of exosomes was quantitated using dynamic light scattering with a Nanosizer^TM^ instrument (Malvern Instruments, UK) [75]. Transmission electron microscopy (TEM) [76] was performed on whole mounts of isolated exosomes using 2.5% glutaraldehyde, postfixed with 1% osmium tetroxide, embedded and stained with uranyl acetate and lead citrate, and observed using a TEM microscope (JEM-1230; JEOL, Japan). Purified exosomes were further characterized by western blot using anti-CD63 and anti-CD81 antibodies [73], as stated below.

### Intravitreal injection

MSCs at the first passage were collected and suspended in PBS at 1 × 10^7^ cells/ml and were injected at 1 μl into each vitreous chamber with a 33-G Hamilton syringe (Hamilton Company, USA) at 6 h after MNU treatment or at 2-week old of *Pde6b*^*mut*^ mice. The collected exosomes were adjusted to a protein concentration of 1 μg/μl and were also injected at 1 μl into each vitreous chamber at 6 h after MNU treatment or at 2-week old of *Pde6b*^*mut*^ mice. For the intravitreal delivery, the injection site was selected just posterior to the limbus and the needle was injected under anesthesia (100 mg/kg ketamine plus 20 mg/kg xylazine intraperitoneally), which was retracted after 2 min to minimize backflow [17].

### Retinal histology and morphology

At sacrifice, retinal tissues were rapidly isolated, fixed overnight with 4% paraformaldehyde, and embedded in paraffin. 5-μm serial sections were prepared (RM2125; Leica, Germany) and underwent H&E staining for tissue histology and morphology, according to previous reports [70]. Quantification of ONL thickness was determined using the ImageJ 1.47 software along the vertical meridian of the eyeball from nasal to temporal side and through the optic nerve head (ONH) [77], and statistically analyzed as area under the curve (AUC).

### TUNEL and IF staining

At sacrifice, retinal tissues were rapidly isolated, fixed in 4% paraformaldehyde, cryoprotected with 30% sucrose, and embedded in the optimal cutting temperature (OCT) compound. The specimens were snap-frozen and sectioned into 15-μm sagittal sections (CM1950; Leica, Germany). Sections were then underwent TUNEL assay using DeadEnd^TM^ Fluorometric TUNEL System according to the manufacturers’ instructions (Promega, USA), counterstained by Hoechst 33342 (Sigma-Aldrich, USA) [32]. The images were further analyzed using the ImageJ 1.47 software from at least five consecutive microscopic fields for TUNEL^+^ cells over total ONL cells. For IF analyses on cone photoreceptors, sections were blocked with 5% bovine serum albumin (BSA) (Sigma-Aldrich, USA) dissolved in PBS for 1 h at room temperature, and stained with a rabbit anti-mouse S-opsin primary antibody (ABN1660; Millipore, USA) overnight at 4 °C at a concentration of 1:1000. After washing with PBS, sections were stained with an AF647-conjugated goat-anti-rabbit secondary antibody for 1 h at room temperature, and were counterstained with Hoechst 33342 (Sigma-Aldrich, USA). Quantification of the percentages of positively stained area over total retinal fields was performed using the ImageJ 1.47 software.

### ERG analysis

The UTAS Visual Diagnostic System with a Big Shot Ganzfeld (LKC Technologies, USA) was employed. Mice were dark-adapted overnight before ERG analysis. Under dim red light conditions, anesthesia was induced by an intraperitoneal injection of 80 mg/kg ketamine and 10 mg/kg chlorpromazine, and mice were then lightly secured to a stage to ensure a stable position for recording. Cornea was anesthetized with a drop of 0.5% proxymetacaine and was kept moist with physiological saline. Platinum circellus record electrodes were placed on each cornea, while reference and ground electrodes were respectively located in the mouth and inserted in the tail. White flashes with the intensity of 3.0 cd.s/m^2^ were applied for stimulating the responses. The band-pass (1–300 Hz) was used to amplify the recorded signals. The line noise was wiped off by a 50-Hz notch filter. Totally 10 scotopic (dark-adapted, mixed rod- and cone-mediated) and 60 photopic (light-adapted, cone-mediated) responses were recorded for analyzing the amplitudes of a-wave and/or b-wave by ERG View v4.380 R software [78].

### Transfection of microRNA mimics, siRNA, and plasmids

The mimics and NC of miR-21, and the siRNA and NC of Pdcd4 were designed by RiboBio (Guangzhou, China) and were transfected into cells at final concentrations of 50 nM, according to previous studies [42, 43]. The Lipofectamine 2000 reagent (Invitrogen, USA) was used for transfection according to the manufacturer’s recommendation. CD63-EGFP plasmids were purchased from Hanheng (Shanghai, China) and were also transfected into MSCs using the Lipofectamine 2000 reagent (Invitrogen, USA). The transfected MSCs or 661W cells were further used for downstream experiments after 48 h incubation.

### In vitro apoptosis assay

661W cells were harvested and evaluated by FITC-conjugated Annexin V and propidium iodide (PI) double staining according to the manufacturer’s instruction of Annexin V Apoptosis Detection Kit I (BD Biosciences, USA). Cell apoptosis was detected with a flow cytometer (CytoFLEX; Beckman Coulter, USA) equipped with the CXP 2.1 software. The percentages of early apoptotic (FITC^+^PI^−^) plus late apoptotic (FITC^+^PI^+^) cells were expressed as apoptotic percentages [55, 79].

### Tracing of MSCs, exosomes, and miR-21

For tracing of MSCs and exosomes, PKH26 (Sigma-Aldrich, USA) was used according to manufacturer’s instructions [73]. For biodistribution of MSCs in the eye, freshly dissected eyes were scanned using the Xenogen IVIS imaging system (Xenogen IVIS Lumina II; PerkinElmer, USA) at 6 h after injection of PKH26-labeled MSCs. For in vivo tracing in the retina, retinal tissues were harvested at 24 h after injection of PKH26-labeled MSCs or exosomes. CD63-EGFP plasmid-transfected MSCs were also injected for in vivo examination of exosome release. For tracing of MSC-derived miR-21 in vivo, Cy5-labeled miR-21 mimics (RiboBio, China) was transfected into MSCs using the Lipofectamine 2000 reagent (Invitrogen, USA) before injection. The retina were then fixed in 4% paraformaldehyde, cryoprotected with 30% sucrose, and embedded in the OCT compound. The specimens were snap-frozen and sectioned into 15-μm sagittal sections (CM1950; Leica, Germany) and counterstained by Hoechst 33342 (Sigma-Aldrich, USA). For in vitro tracing, 661W cells were treated by PKH26-labeled exosomes for 48 h, fixed in 4% paraformaldehyde, blocked by 5% BSA, and immunostained with a rabbit anti-mouse primary antibody at a concentration of 1:100 for β-tubulin (ab18207; Abcam, UK) overnight at 4 °C. After washing with PBS, the sections were stained with a FITC-conjugated goat-anti-rabbit secondary antibody and counterstained by Hoechst 33342 (Sigma-Aldrich, USA). For tracing of MSC-derived miR-21 into 661W cells in vitro, Cy3-labeled miR-21 mimics (RiboBio, China) was transfected into MSCs using the Lipofectamine 2000 reagent (Invitrogen, USA) before MSCs being added in the upper chamber of the Transwell system. MSCs and 661W cells were then co-cultured for 48 h, and 661W cells were fixed in 4% paraformaldehyde and counterstained by Hoechst 33342 (Sigma-Aldrich, USA).

### qRT-PCR

Total RNA was extracted from retinal tissues or cells by direct adding of Trizol Reagent (Takara, Japan) and purified by phenol–chloroform extraction. For mRNA, cDNA synthesis was performed using the PrimeScript^TM^ RT Reagent Kit (Takara, Japan), and the primer sequences of the genes detected in this study were listed in the Supplementary Information (Table S1). For microRNA, reverse transcription primers and qRT-PCR primers were designed by RiboBio (Guangzhou, China) as the Bulge-loop^TM^ miRNA qRT-PCR Primer Sets. qRT-PCR detection was performed using the SYBR Premix Ex Taq II Kit (Takara, Japan) by a Real-Time System (CFX96; Bio-Rad, USA). The relative expression level of each gene was obtained by the cycle number after normalizing against *Glyceraldehyde-3-phosphate dehydrogenase* (*Gapdh*, for mRNA) and *RNU6* (for microRNA) abundances using the 2^−ΔΔCT^ method [42, 43].

### Western blot

Traditional western blot was performed as previously described [79]. Whole lysates of exosomes were prepared using the Lysis Buffer (Beyotime, China). Proteins were extracted, loaded on sodium dodecyl sulfate-polyacrylamide gels, transferred to polyvinylidene fluoride membranes (Millipore, USA), and blocked with 5% BSA (Sigma-Aldrich, USA) in PBS with 0.1% Tween for 2 h in room temperature. The membranes were incubated overnight at 4 °C with the following primary antibodies: a rabbit anti-mouse primary antibody at a concentration of 1:1000 for CD63 (sc-5275; Santa Cruz Biotechnology, USA); and a rabbit anti-mouse primary antibody at a concentration of 1:1000 for CD81 (sc-70803; Santa Cruz Biotechnology, USA). The membranes were then incubated with peroxidase-conjugated goat anti-rabbit secondary antibodies at a concentration of 1:40000 for 1 h in room temperature. The blotted bands were visualized using an enhanced chemiluminescence Kit (Amersham Biosciences, USA) and a gel imaging system (5500; Tanon, China).

Capillary-based immunoassay was performed for retinal and 661W cell lysates using the Wes-Simple Western method with the anti-rabbit detection module (ProteinSimple, USA) [80]. Protein expression was measured by chemiluminescence and quantified as AUC using the Compass for Simple Western program (ProteinSimple, USA). Proteins were detected with the following primary antibodies: a rabbit anti-mouse primary antibody for Pdcd4 (9535S; Cell Signaling Technology, USA) and a rabbit anti-mouse primary antibody for Gapdh (5174S; Cell Signaling Technology, USA) at concentrations of 1:1000 (both from Cell Signaling Technology, USA).

### Sample processing

Each experiment was performed in at least three independent times and data represented biological replicates. Sample size of animals was determined based on preliminary tests for variation to ensure adequate power for analysis. Samples were blindly allocated to each of the experimental group based on randomization and were also blind for data analysis. No samples were excluded from the results.

### Statistical analysis

The results are represented as median ± range or as box (25th, 50th, and 75th percentiles) and whisker (range) plots for indicated experiments. The data were analyzed using non-parametric methods, i.e. the Mann–Whitney *U* tests for two group comparisons or the Kruskal–Wallis tests for multiple group comparisons, in the GraphPad Prism 5.01 software for not meeting normal distribution. Values of *P* < 0.05 were considered to be statistically significant.

## Supplementary information

Supplementary Figure Legends

Supplementary Table

Figure S1

Figure S2

Figure S3

Figure S4

Figure S5

Figure S6
