# Supplementary material for: Photoreceptor protection by mesenchymal stem cell transplantation identifies exosomal MiR-21 as a therapeutic for retinal degeneration
Source: Cell Death Differ. 2020 Oct 20;28(3):1041–61. doi: 10.1038/s41418-020-00636-4 (PMC7937676; doi:10.1038/s41418-020-00636-4)
Supplement: Supplementary file 1 — Supplementary Figure Legends [file 41418_2020_636_MOESM1_ESM.docx]

**Supplementary figure legends**

**Figure S1.** Identification of mesenchymal stem cells (MSCs) and exosomes (EXO) used in this study.

(A) Flow cytometric analysis of surface antigens of MSCs. Sca1, stem cell antigen 1.

(B) Osteogenic differentiation of MSCs *in vitro* exhibited by Alizarin red staining. Scale bar=500 μm.

(C) Adipogenic differentiation of MSCs *in vitro* exhibited by Oil red O staining. Scale bar=500 μm.

(D) Transmission electron microscopy (TEM) analysis of EXO. Scale bar=200 nm.

(E) Diameter of EXO analyzed by dynamic light scattering.

(F) Western blot analysis of markers of EXO compared to MSCs.

(G) Quantification of exosomal protein in MSCs after treatments with dimethyl sulfoxide (DMSO) and GW4869. *, *P* < 0.05 by the Kruskal-Wallis test. *N* = 4 per group.

(H) Tracing of PKH26-labeled MSCs (red) in the retina tissue counterstained by Hoechst 33342 (blue) after intravitreal injection for 24 h. MNU, *N*-methyl-*N*-nitrosourea; MSCT, mesenchymal stem cell transplantation; INL, inner nuclear layer; ONL, outer nuclear layer; NC, negative control, injection of MSCs without staining. Scale bar=50 μm.

(I) Viability of MSCs *in vitro* examined by methyl thiazolyl tetrazolium (MTT) after treatments with DMSO and GW4869. NS, not significant, *P* > 0.05 by the Mann-Whitney *U* test. *N* = 6 per group.

(J) Osteogenic differentiation of MSCs *in vitro* exhibited by Alizarin red staining after treatments with DMSO and GW4869. Scale bar=500 μm.

(K) Quantification of MSC mineralization *in vitro*. NS, not significant, *P* > 0.05 by the Mann-Whitney *U* test. *N* = 3 per group.

(L) Quantitative real time polymerase chain reaction (qRT-PCR) analysis of expression levels of *miR-21* in MSC-derived EXO, normalized to *Rnu6*. WT, wild type. ND, not detected. *N* = 3 per group.

Data are represented as box (25^th^, 50^th^ and 75^th^ percentiles) and whisker (range) plots for (G) and (I). Data represent median ± range for (K) and (L).

**Figure S2.** Exosomes mediates therapeutic effects of mesenchymal stem cell transplantation (MSCT) on *N*-methyl-*N*-nitrosourea (MNU)-induced retinal degeneration.

(A, B) Representative hematoxylin and eosin (H&E) staining images of retinal tissues (A) and the corresponding quantitative analysis of outer nuclear layer (ONL) thickness (B). Ctrl, control; GCL, ganglion cell layer; INL, inner nuclear layer; ONH, optic nerve head. Scale bars=50 μm. *, *P* < 0.05 by the Kruskal-Wallis test for area under curve (AUC). *N* = 3 per group.

(C-E) Representative scotopic electroretinography (ERG) waveforms (C) and the corresponding quantitative analyses of amplitude changes of a-wave (D) and b-wave (E). *, *P* < 0.05 by the Kruskal-Wallis tests. *N* = 6 per group.

(F, G) Representative photopic ERG waveforms (F) and the corresponding quantitative analysis of b-wave amplitude changes (G). *, *P* < 0.05 by the Kruskal-Wallis tests. *N* = 6 per group.

(H, I) Representative H&E staining images of retinal tissues (H) and the corresponding quantitative analysis of ONL thickness (I). EXOT, exosomal transplantation after MNU injection. Scale bars=50 μm. *, *P* < 0.05 by the Kruskal-Wallis test for AUC. *N* = 3 per group.

(J-L) Representative scotopic ERG waveforms recorded for retinal functional analysis (J) and the corresponding quantitative analyses of amplitude changes of a-wave (K) and b-wave (L). *, *P* < 0.05 by the Kruskal-Wallis tests. *N* = 6 per group.

(M, N) Representative photopic ERG waveforms (M) and the corresponding quantitative analysis of b-wave amplitude changes (N). *, *P* < 0.05 by the Kruskal-Wallis tests. *N* = 6 per group.

(O) Representative immunofluorescent (IF) staining images of retinal tissues showing cone photoreceptor bodies (white) counterstained by Hoechst 33342 (blue). Scale bars=25 μm.

Data represent median ± range for (B) and (I). Data are represented as box (25^th^, 50^th^ and 75^th^ percentiles) and whisker (range) plots for (D), (E), (G), (K), (L) and (N).

**Figure S3.** Blockade of exosomal generation by GW4869 inhibits therapeutic effects of mesenchymal stem cell transplantation (MSCT) on *N*-methyl-*N*-nitrosourea (MNU)-induced retinal degeneration.

(A, B) Representative hematoxylin and eosin (H&E) staining images of retinal tissues (A) and the corresponding quantitative analysis of outer nuclear layer (ONL) thickness (B). Mice were injected with MNU with or without MSCT. GW4869, a neutral sphingomyelinase inhibitor for blocking exosome generation, which was used for MSC preconditioning before transplantation. GCL, ganglion cell layer; INL, inner nuclear layer; ONH, optic nerve head. Scale bars=50 μm. *, *P* < 0.05 by the Kruskal-Wallis test for area under curve (AUC). *N* = 3 per group.

(C-E) Representative scotopic electroretinography (ERG) waveforms recorded for retinal functional analysis (C) and the corresponding quantitative analyses of amplitude changes of a-wave (D) and b-wave (E). *, *P* < 0.05 by the Kruskal-Wallis tests. *N* = 6 per group.

(F, G) Representative photopic ERG waveforms (F) and the corresponding quantitative analysis of b-wave amplitude changes (G). *, *P* < 0.05 by the Kruskal-Wallis tests. *N* = 6 per group.

Data represent median ± range for (B). Data are represented as box (25^th^, 50^th^ and 75^th^ percentiles) and whisker (range) plots for (D), (E) and (G).

**Figure S4.** Exosomal transplantation (EXOT) protects miR-21-deficient (miR-21^-/-^) mice against *N*-methyl-*N*-nitrosourea (MNU)-induced retinal degeneration.

(A, B) Representative hematoxylin and eosin (H&E) staining images of retinal tissues (A) and the corresponding quantitative analysis of outer nuclear layer (ONL) thickness (B). Ctrl, control; EXOT, transplantation of mesenchymal stem cell (MSC)-derived exosomes after MNU injection; GCL, ganglion cell layer; INL, inner nuclear layer; ONH, optic nerve head. Scale bars=50 μm. *, *P* < 0.05 by the Kruskal-Wallis test for area under curve (AUC). *N* = 3 per group.

(C-E) Representative scotopic electroretinography (ERG) waveforms (C) and the corresponding quantitative analyses of amplitude changes of a-wave (D) and b-wave (E). *, *P* < 0.05 by the Kruskal-Wallis tests. *N* = 6 per group.

(F, G) Representative photopic ERG waveforms (F) and the corresponding quantitative analysis of b-wave amplitude changes (G). *, *P* < 0.05 by the Kruskal-Wallis tests. *N* = 6 per group.

(H, I) Representative terminal deoxynucleotidyl transferase dUTP nick end labeling (TUNEL, green) staining images of retinal tissues counterstained by Hoechst 33342 (blue) (H) and the corresponding quantitative analysis of percentages of TUNEL^+^ cells over total ONL cells (I). Scale bars=50 μm. *, *P* < 0.05 by the Mann-Whitney *U* test. *N* = 4 per group.

Data represent median ± range for (B). Data are represented as box (25^th^, 50^th^ and 75^th^ percentiles) and whisker (range) plots for (D), (E), (G) and (I).

**Figure S5.** Original bands obtained from the digital signals using the Wes-Simple Western method.

(A) Related to Figure 7D.

(B) Related to Figure 7G.

(C) Related to Figure 7I.

(D) Related to Figure 7K.

**Figure S6.** Exosomal miR-21 counteracts *N*-methyl-*N*-nitrosourea (MNU)-induced retinal degeneration.

(A, B) Representative hematoxylin and eosin (H&E) staining images of retinal tissues (A) and the corresponding quantitative analysis of outer nuclear layer (ONL) thickness (B). WT-EXOT, transplantation of exosomes derived from wild-type mesenchymal stem cells (MSCs) after MNU injection; miR-21^-/-^-EXOT, transplantation of exosomes derived from miR-21-deficient MSCs after MNU injection; GCL, ganglion cell layer; INL, inner nuclear layer; ONH, optic nerve head. Scale bars=50 μm. *, *P* < 0.05 by the Kruskal-Wallis test for area under curve (AUC). *N* = 3 per group.

(C-E) Representative scotopic electroretinography (ERG) waveforms (C) and the corresponding quantitative analyses of amplitude changes of a-wave (D) and b-wave (E). *, *P* < 0.05 by the Kruskal-Wallis tests. *N* = 6 per group.

(F, G) Representative photopic ERG waveforms (F) and the corresponding quantitative analysis of b-wave amplitude changes (G). *, *P* < 0.05 by the Kruskal-Wallis tests. *N* = 6 per group.

(H, I) Representative H&E staining images of retinal tissues (H) and the corresponding quantitative analysis of ONL thickness (I). Ctrl, control; NC, negative control of miR-21 mimics. MNU-injected mice were transplanted with exosomes derived from miR-21-deficient MSCs, which were transfected with or without NC or miR-21 mimics. Scale bars=50 μm. *, *P* < 0.05 by the Kruskal-Wallis test for AUC. *N* = 3 per group.

(J-L) Representative scotopic ERG waveforms recorded for retinal functional analysis (J) and the corresponding quantitative analyses of amplitude changes of a-wave (K) and b-wave (L). *, *P* < 0.05 by the Kruskal-Wallis tests. *N* = 6 per group.

(M, N) Representative photopic ERG waveforms (M) and the corresponding quantitative analysis of b-wave amplitude changes (N). *, *P* < 0.05 by the Kruskal-Wallis tests. *N* = 6 per group.

Data represent median ± range for (B) and (I). Data are represented as box (25^th^, 50^th^ and 75^th^ percentiles) and whisker (range) plots for (D), (E), (G), (K), (L) and (N).
