# Supplementary material for: Photoreceptor protection by mesenchymal stem cell transplantation identifies exosomal MiR-21 as a therapeutic for retinal degeneration
Source: Cell Death Differ. 2020 Oct 20;28(3):1041–61. doi: 10.1038/s41418-020-00636-4 (PMC7937676; doi:10.1038/s41418-020-00636-4)
Supplement: Supplementary file 2 — Supplementary Table [file 41418_2020_636_MOESM2_ESM.docx]

**Supplementary table**

**Table S1.** Primer sequences for mRNAs in the present study.

| **Gene** | **Primer sequences** |
| --- | --- |
| *Gapdh* | Forward: 5’-TGTGTCCGTCGTGGATCTGA-3’  Reverse: 5’-TTGCTGTTGAAGTCGCAGGAG-3’ |
| *Pdcd4* | Forward: 5’-GCCAAAGAAAGGTGGTGCAG-3’  Reverse: 5’-AAATGCGGTCTCATCCAGGG-3’ |
| *Smad5* | Forward: 5’-TGGATCTAAGCAAAAGGAAGTTTGT-3’  Reverse: 5’-GGCATGTGCGGTTCATTGTG-3’ |
| *Spry1* | Forward: 5’-AAGCCATCAGAGGCAAT-3’  Reverse: 5’-TTTTCGGGTCTTGGTGCAGT-3’ |
| *Spry2* | Forward: 5’-TGAAAGACTCCCACGGTCTGC-3’  Reverse: 5’-AGCTGACAGTGCTGATGGAC-3’ |
| *Tfap2a* | Forward: 5’-TGGGAACCTGGAAGCTTGTC-3’  Reverse: 5’-CTTTCCTTGTGGCAAAGCCC-3’ |
