# Supplementary figures and images for: Photoreceptor protection by mesenchymal stem cell transplantation identifies exosomal MiR-21 as a therapeutic for retinal degeneration
Source: Cell Death Differ. 2020 Oct 20;28(3):1041–61. doi: 10.1038/s41418-020-00636-4 (PMC7937676; doi:10.1038/s41418-020-00636-4)

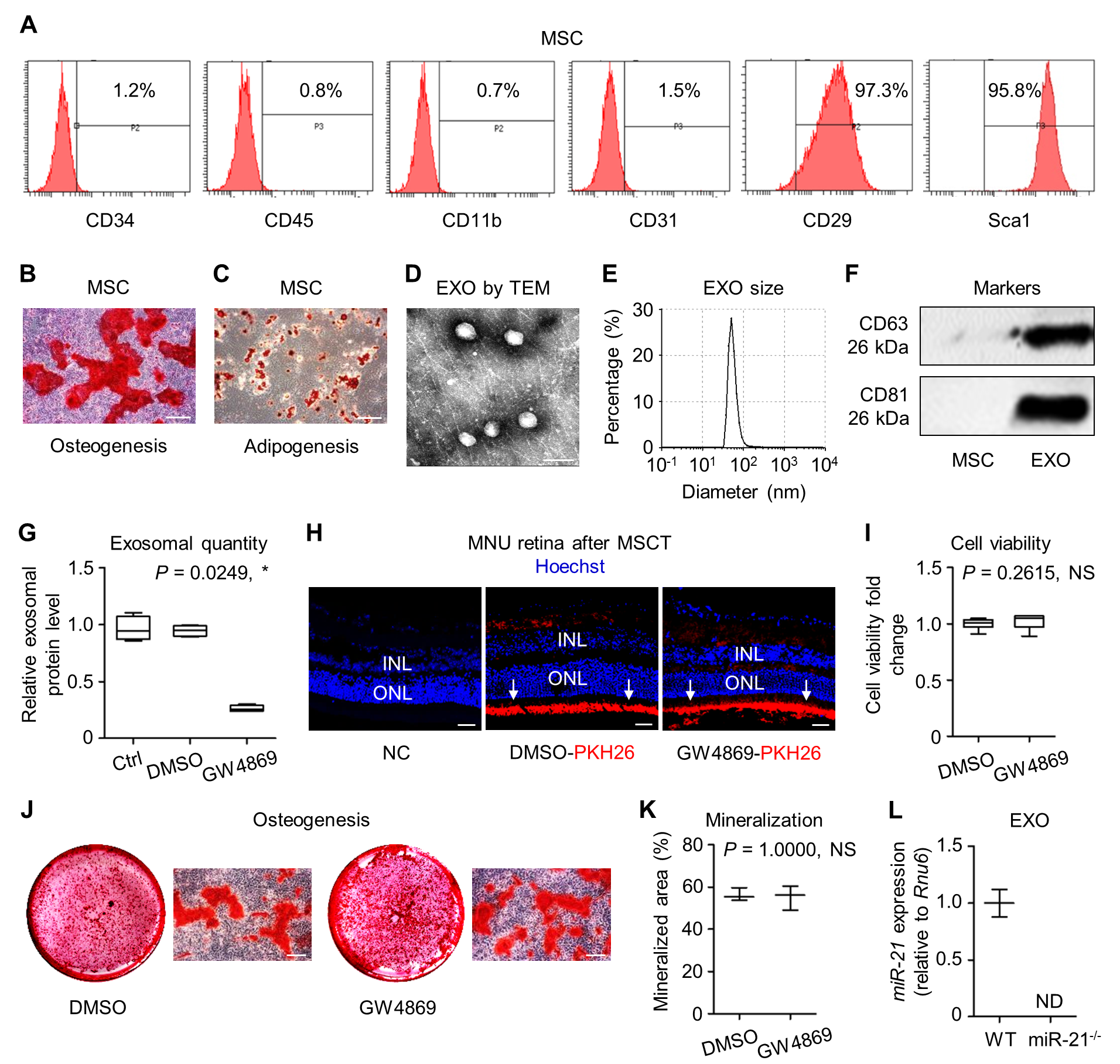

Supplement: Supplementary file 3 — Figure S1 [file 41418_2020_636_MOESM3_ESM.tif]

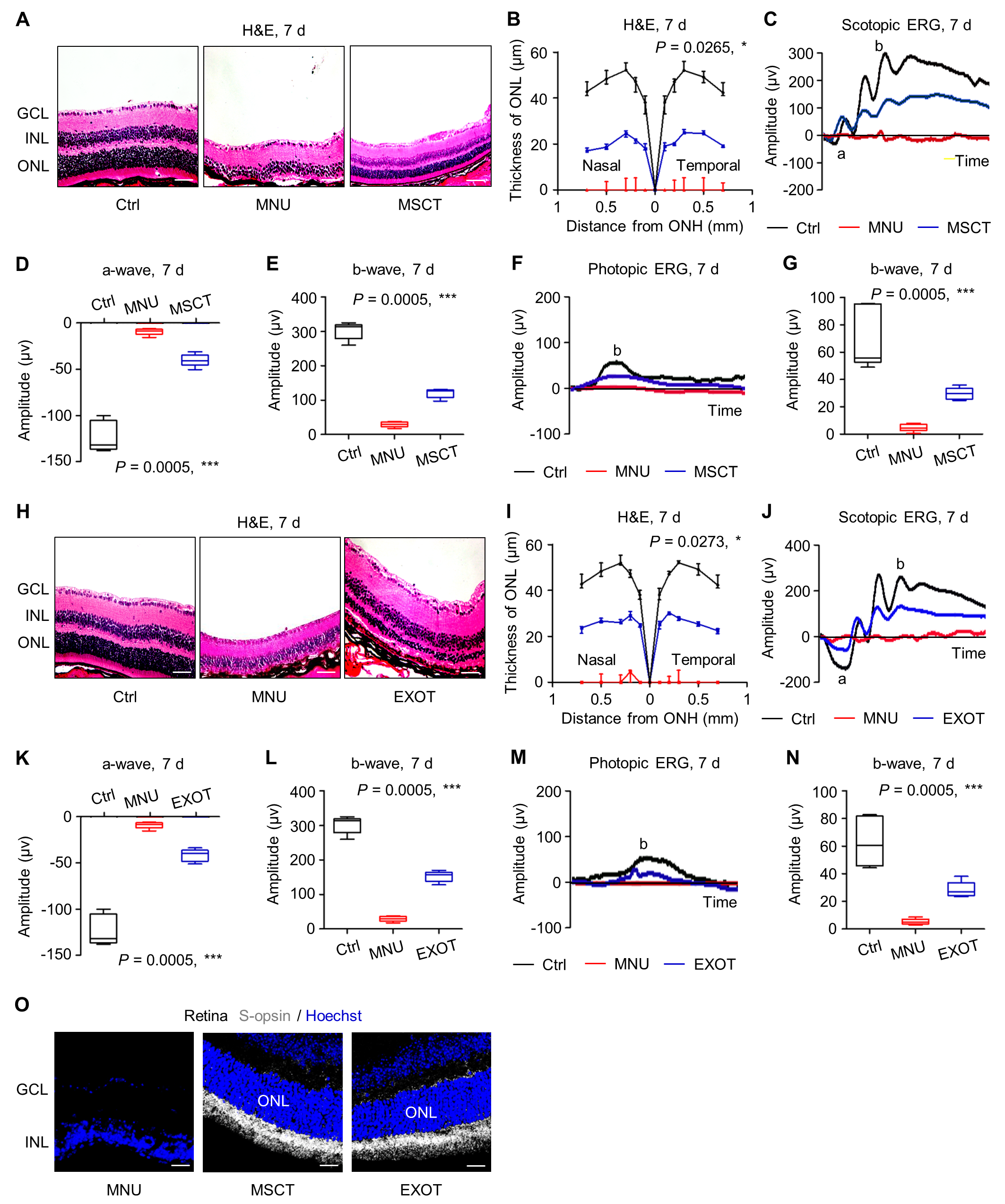

Supplement: Supplementary file 4 — Figure S2 [file 41418_2020_636_MOESM4_ESM.tif]

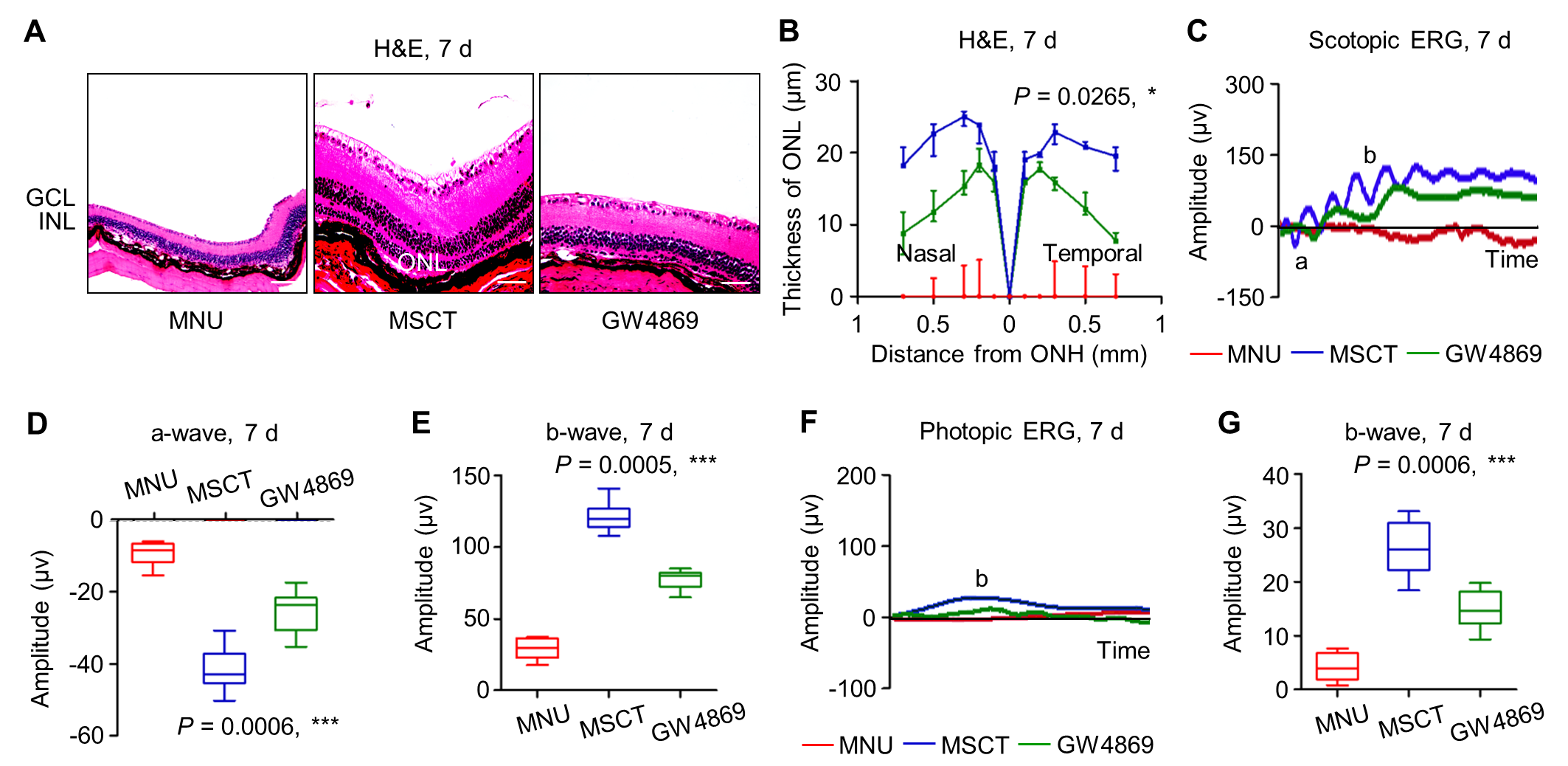

Supplement: Supplementary file 5 — Figure S3 [file 41418_2020_636_MOESM5_ESM.tif]

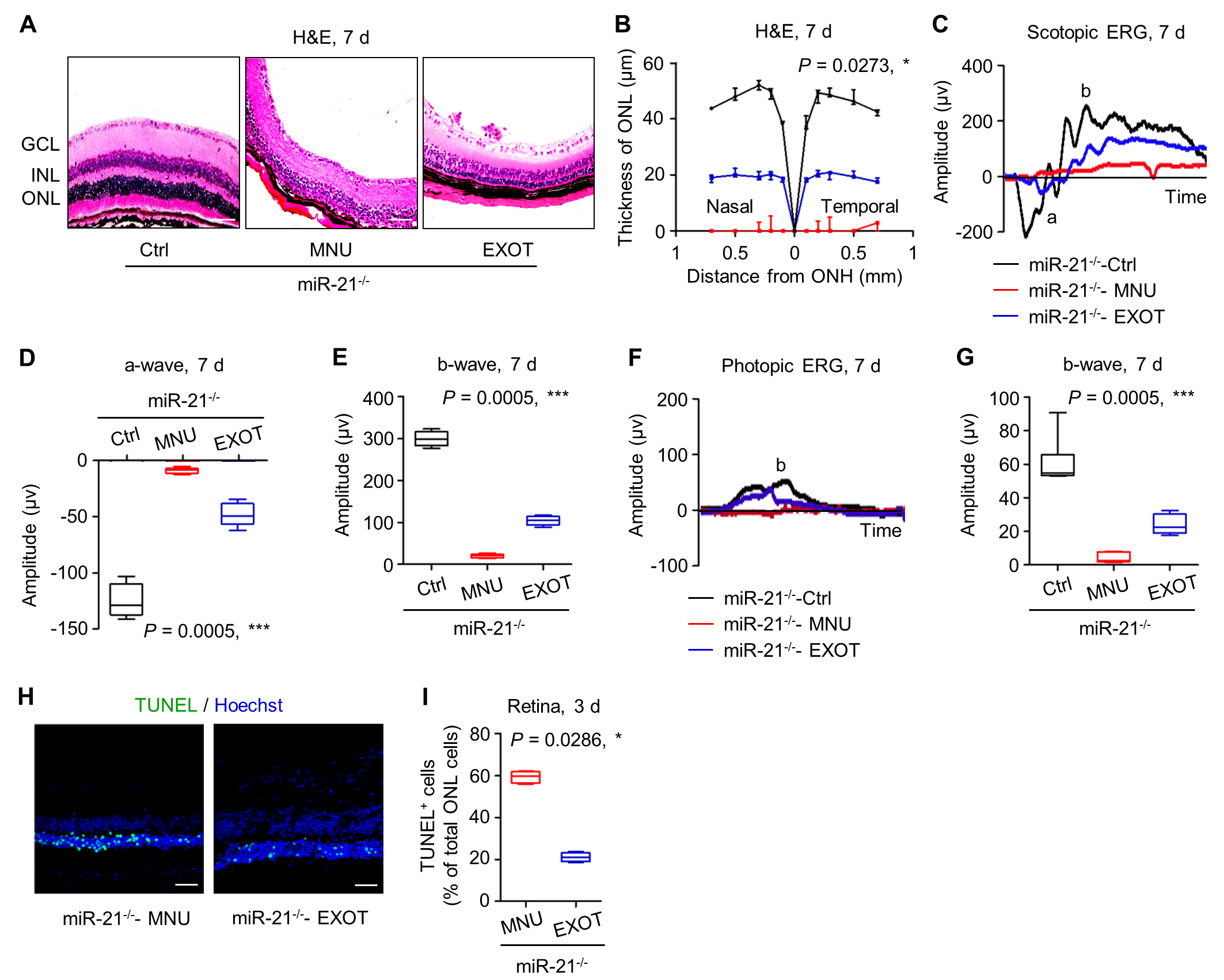

Supplement: Supplementary file 6 — Figure S4 [file 41418_2020_636_MOESM6_ESM.tif]

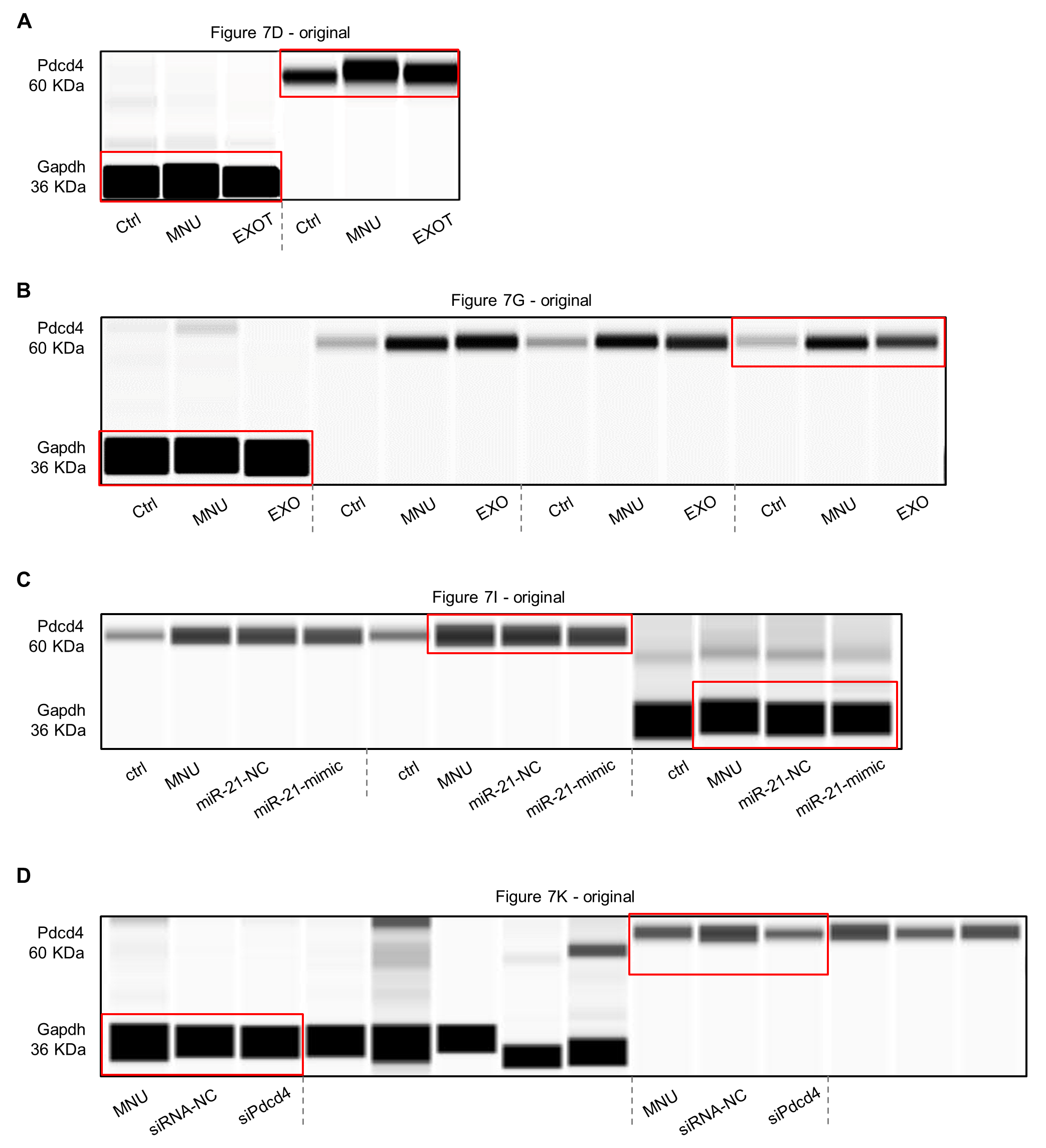

Supplement: Supplementary file 7 — Figure S5 [file 41418_2020_636_MOESM7_ESM.tif]

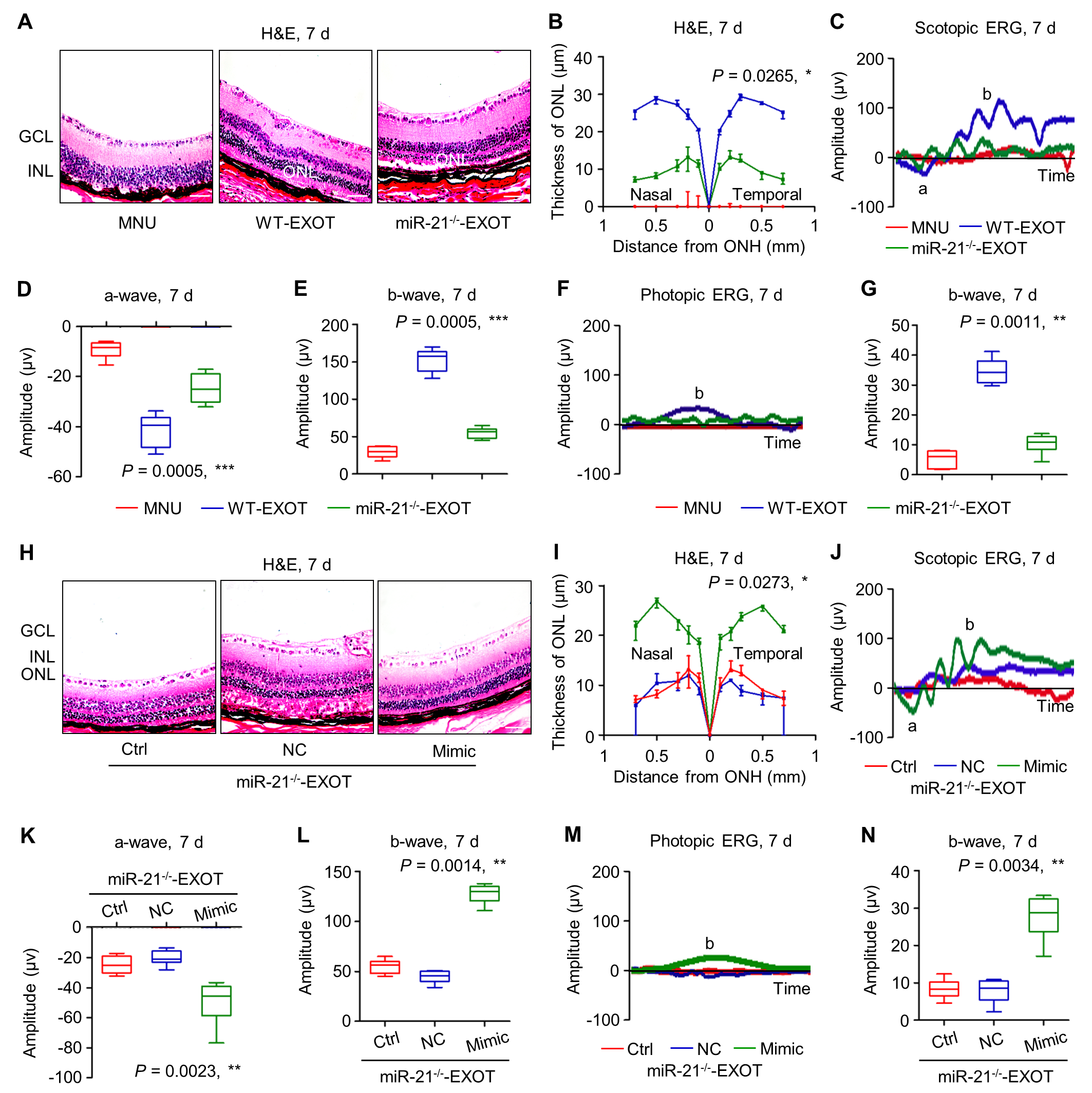

Supplement: Supplementary file 8 — Figure S6 [file 41418_2020_636_MOESM8_ESM.tif]
